# Overcoming Physiological Barriers to Nanoparticle Delivery—Are We There Yet?

**DOI:** 10.3389/fbioe.2019.00415

**Published:** 2019-12-17

**Authors:** Oliver S. Thomas, Wilfried Weber

**Affiliations:** ^1^Faculty of Biology, University of Freiburg, Freiburg, Germany; ^2^Signalling Research Centres BIOSS and CIBSS, University of Freiburg, Freiburg, Germany; ^3^Spemann Graduate School of Biology and Medicine, University of Freiburg, Freiburg, Germany

**Keywords:** nanomedicine, nanoparticle, nanocarrier, drug delivery, barrier, EPR effect, stimulus-responsive, PEG

## Abstract

The exploitation of nanosized materials for the delivery of therapeutic agents is already a clinical reality and still holds unrealized potential for the treatment of a variety of diseases. This review discusses physiological barriers a nanocarrier must overcome in order to reach its target, with an emphasis on cancer nanomedicine. Stages of delivery include residence in the blood stream, passive accumulation by virtue of the enhanced permeability and retention effect, diffusion within the tumor lesion, cellular uptake, and arrival at the site of action. We also briefly outline strategies for engineering nanoparticles to more efficiently overcome these challenges: Increasing circulation half-life by shielding with hydrophilic polymers, such as PEG, the limitations of PEG and potential alternatives, targeting and controlled activation approaches. Future developments in these areas will allow us to harness the full potential of nanomedicine.

## 1. Introduction

**Figure 1:**
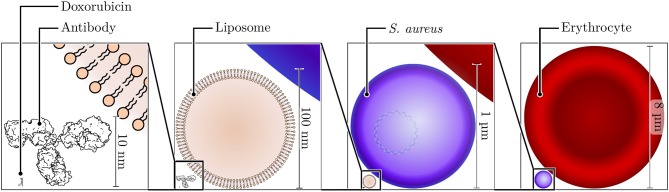
Size comparison of nano- and microscale structures. Shown to the bottom left is the skeleton structure of doxorubicin, a typical cargo molecule for nanoparticle formulations, with a size of ~1.5 nm. Adjacent to this small molecule, a typical antibody molecule (height of ~10 nm) is shown next to a small unilamellar liposome with a diameter of 100 nm. The diameter of the bacterium *S. aureus* is ~1 μm, and that of a human erythrocyte is ~8 μm.

In the ever-continuing arms race between medical researchers and the ailments they are trying to tackle, nanotechnology has emerged as a useful ally. A nanoparticle (NP) is an object with dimensions in the nanometer range (Figure 1). A nanocarrier is a nanoparticle utilized for the transport of a cargo, for instance a therapeutic molecule. The diversity of available nanoparticles for drug delivery is considerable and includes polymeric nanoparticles, dendrimers, carbon nanotubes, quantum dots, metallic nanoparticles or lipid-based systems, such as micelles or liposomes (Hughes, 2005; Cho et al., 2008; Bogart et al., 2014; Matea et al., 2017). Liposomes were first described by Bangham et al. (1965) and would eventually prove to be a promising candidate for the encapsulation of therapeutic molecules. In fact, the first nanoparticulate drug to be approved by the US Food and Drug administration (FDA) has been Doxil (marketed as Caelyx outside the US) (Barenholz, 2012), a liposomal formulation of the cytostatic agent doxorubicin. Several liposomal drug products are now in clinical use (Bulbake et al., 2017), many of which are intended for the treatment of cancer, and new ones continue to reach approval status. In 2015, the FDA approved Onivyde (liposomal irinotecan) for the use in metastatic pancreatic cancer, a disease with a dismal prognosis for affected patients. The phase 3 trial showed an (albeit moderate) extension of overall survival (Wang-Gillam et al., 2016) that was confirmed in a recent follow-up study (Wang-Gillam et al., 2019). In 2017, Vyxeos (liposomal synergistic combination of daunorubicin and cytarabin) was approved for acute myeloid leukemia (Lancet et al., 2018). Innovative liposomal formulations also make their mark in disciplines other than oncology: Arikayce (liposomal amikacin) was FDA-approved in 2018 for the management of non-tuberculous mycobacteria infection (Griffith et al., 2018)—however, the application in Europe was withdrawn in 2016 (with intent to resubmit) after data from a phase 2 trial failed to convince the European Medicines Agency (EMA) of the drug's benefits. Also in 2018, a vaccine was approved for the prevention of Herpes Zoster in older patients, which contains a liposomally formulated adjuvant (Shingrix).

These examples highlight the potential of nanoparticulate formulations in general, and liposomally encapsulated drugs in particular. They also illustrate the breadth of applications (potential and actual) for these types of therapeutics, which is supported by an exhaustive overview of nanoparticles either approved clinically or undergoing clinical trials (Anselmo and Mitragotri, 2016, 2019).

This review aims to highlight the challenges faced by such formulations during their journey toward their destination and what strategies have been devised to try and circumvent these obstacles, with a focus on cancer therapy. Previous excellent reviews have considered related issues. For instance, Blanco et al. reviewed biological barriers to nanoparticle delivery, highlighting the influence of the physicochemical and geometric properties of nanoparticles (Blanco et al., 2015). Yu et al. considered numerous nano-scaled delivery devices with a focus on protein delivery and topical delivery modalities (Yu et al., 2016). This work is supposed to complement them with recent findings and developments of the last years. In particular, important progress has been made in attempts to quantitatively understand the processes leading to nanoparticle delivery and internalization. When examples are given for principles of nanoparticle design, we furthermore focused on systems which were efficacious clinically or at least in mammalian model organisms (as opposed to cell culture assays alone), whenever possible.

To illustrate the underlying principles, we will follow an injected nanoparticle from the site of injection toward the site of action. We first summarize the basis of the enhanced permeability and retention (EPR) effect and highlight its heterogeneous nature. We then shift the focus from the physiology of the disease to the characteristics of the nanoparticle and discuss shielding strategies, which are required to confer long half-lives on nanoparticles in order to exploit the EPR effect and allow arrival at the tumor. Furthermore, we consider options for stimulus-responsive designs of nanocarriers to maximize their capability of reaching (and interacting with) their target cells. Finally, we give an overview about targeting modalities to direct nanoparticles to their destined target cells within the tumor tissue and their intracellular sites of action.

## 2. Cancer Nanomedicine: From Injection to Tumor

A large amount of effort is being expended to enable and advance the application of nanotechnology-based drugs for the treatment of cancer. To exert their intended effect and eliminate malignant cells, these agents, like any drug, must first and foremost be capable of reaching the site of the lesion. A frequently cited, yet controversially discussed concept in research aimed at developing new nanocarriers for oncological treatments is the so-called enhanced permeability and retention (EPR) effect (Rosenblum et al., 2018). The term was coined by Matsumura and Maeda (1986) and describes the tendency of macromolecules and nano-sized-particles to accumulate in neoplastic tissues, therefore facilitating passive targeting without the need for additional modifications of the carrier.

### 2.1. The Pathophysiological Basis of the EPR Effect

The underlying fundamental process toward the establishment of the EPR effect is neovascularization of the tumor tissue, an occurrence that was labeled as one of the hallmarks of cancer (Hanahan and Weinberg, 2011). It results in the sprouting of new vessels which are, however, of inferior quality compared to healthy vessels. The wall of regular capillaries is primarily made up of endothelial cells, which contain the blood flow toward their luminal side. In most tissues, endothelial cells are connected by tight junctions. In some specialized tissues (such as the kidney glomeruli, endocrine glands or the intestine), the endothelial wall is punctured by fenestrae, small pores of ~60 nm in diameter covered by a negatively charged glycocalyx. The capillaries of the liver and bone marrow feature larger transcellular pores in the endothelial cells, allowing exchange of serum proteins with the interstitium, but this process is highly regulated (Stan, 2007). In the spleen, the capillaries display true intercellular gaps which allows extravasation of erythrocytes and requires them to be deformable enough to re-enter the venous system, filtering out aged and rigid cells (Mebius and Kraal, 2005).

As a tumor continues to grow, its demands increase regarding the acquisition of oxygen and nutrients on the one hand, and the expulsion of waste products on the other. Simultaneously, the distance to the nearest capillary increases. A normoxic environment persists in a radius of ~100 μm around a vessel (Fang et al., 2008), with hypoxia becoming increasingly prevalent as the distance increases further. The hypoxia-inducible factor 1 (HIF-1) is a dimeric transcription factor, consisting of HIF-1α and HIF-1β (Eales et al., 2016). As O_2_ levels decrease, HIF accumulates and induces transcription of its target genes, which includes the vascular endothelial growth factor (VEGF) family. The VEGFs are key players during angiogenesis, but by no means the only one. In tumors, the finely balanced microenvironment of angiogenic factors is disrupted, VEGF is not only upregulated by HIF via hypoxia, but also via the activation of oncogenes (Dvorak, 2003), resulting in aberrant vessels that are highly heterogeneous and differ from normal vessels in several important aspects (Less et al., 1991; Nagy et al., 2010; Azzi et al., 2013).

The induced vessels display gaps in between the endothelial cells (Hashizume et al., 2000) and are less selective regarding the permeability of particles. Around tumor vessels, the sheet of pericytes [a heterogeneous cell type which surrounds healthy vessels and is important for their proper functionality (Armulik et al., 2005; Attwell et al., 2016)] is not necessarily completely absent. However, their association with the endothelial wall is loose and their morphology differs from regular pericytes by the presence of protrusions away from the vessel wall, which are not seen in their regular counterparts (Morikawa et al., 2002). Likewise, the basal membrane of these vessels is compromised and differs in thickness, compactness and its cellular association (Baluk et al., 2003; Kalluri, 2003).

Additionally, tumors are frequently also sites of chronic inflammation to which a diverse array of different leukocytes is recruited (Hanahan and Weinberg, 2011; Coussens et al., 2013). They contribute to the production of tissue mediators of inflammation, which act on blood vessels to increase their permeability, further increasing leakiness, and agents modulating the relevant pathways can be used to modulate the EPR effect (Wu et al., 1998; Maeda et al., 2000).

These irregularities contribute to an inadequate blood supply of the tumor, creating a hypoxic and acidic milieu. They also account for the *enhanced permeability* component of the EPR effect and allow extravasation of macromolecules and particles up to ~400 nm in size due to increased leakiness (Gerlowski and Jain, 1986; Yuan et al., 1995), but depending on the tumor type, this cutoff can be larger or smaller (Hobbs et al., 1998).

The second component, the *enhanced retention*, is a consequence of the aberrant lymphatic architecture (Stacker et al., 2014). Although metastatic spread frequently occurs by means of lymphatic dissemination, this appears to be mediated by lymphatic vessels in the periphery of the tumor mass, whereas internal vessels tend to collapse under the high tissue pressure (Leu et al., 2000; Padera et al., 2002). Consequently, tissue homeostasis within tumors is disrupted, and previously extravasated particles are not efficiently funneled back into the blood via lymphogenic transport through the ductus thoracicus (Noguchi et al., 1998).

Although the effects mentioned above were initially described in a static context, the tumor vasculature and the EPR effect appear to be subject to dynamic changes, as vents within the vessels open transiently to allow efflux of fluid into the surrounding tissues (Matsumoto et al., 2016). For larger particles, these events, termed eruptions, may be the only chance to leave the vessel lumen, and therefore allow them fewer opportunities to re-enter the circulation the way they left it, resulting in their entrapment (Ngoune et al., 2016).

In sum, both aspects (enhanced permeability and enhanced retention) can result in accumulation of particles, given sufficiently long circulation times of the particles in question for this process to take place.

### 2.2. Magnitude and Heterogeneity of the EPR Effect

Cancer is a generic term for the description of a large and heterogeneous class of diseases. The National Cancer Institute lists almost 200 types of cancers on their website. Likewise, the alterations described above are highly heterogeneous, within and between tumors, and the EPR effect cannot simply be generalized as a feature of all cancers (Maeda, 2015; Danhier, 2016). For less well-vascularized lesions, the efficiency of accumulation tends to be higher for small particles, whereas the influence of particle size diminishes as the lesion vascularization and leakiness increase (Cabral et al., 2011).

**Figure 2:**
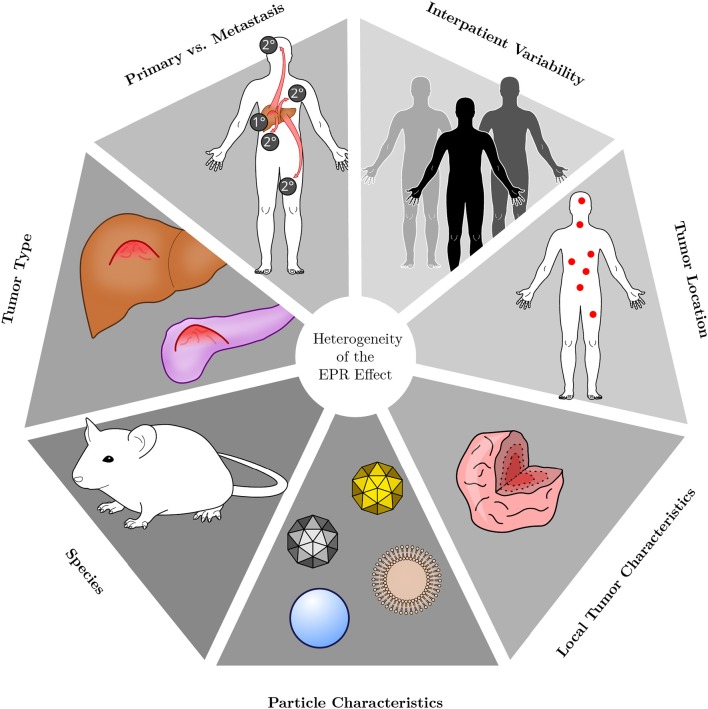
Heterogeneity of the EPR effect. The EPR effect is a very heterogeneous phenomenon. Its presence and magnitude depend on the type of tumor under consideration, whether the lesion is of primary or metastatic origin and on the characteristics of each individual patient. Additionally, in which host tissue a lesion resides and where it is located is influential as well. Within a given tumor, accumulation of nanoparticle therapeutics may be heterogeneous owing to internal tissue composition and characteristics. Different types of particles are likewise heterogeneous in their behavior, owing to variations in, for instance, size, shape, charge or material. Most preclinical research is performed in small rodents, however, the species under investigation will also affect the EPR effect.

Broadly, many different factors influence the unique prevalence of the EPR effect in a given lesion, including the tumor type and stage, the characteristics of the individual patient under consideration, as well as the location of the tumor and local properties in different zones of a tumor. For example, for Kaposi's sarcoma (KS), a sarcoma of vascular origin (Radu and Pantanowitz, 2013), doxorubicin levels in the lesion area were higher after treatment with Doxil compared to non-PEGylated doxorubicin (Northfelt et al., 1996) [but overall survival was not improved (Northfelt et al., 1998; Cooley et al., 2007; Udhrain et al., 2007)]. Contrary to KS, pancreatic adenocarcinomas tend to be hypovascular (Sofuni et al., 2005; Olive et al., 2009), potentially hampering the EPR effect [yet, a combination therapy of Onivyde plus 5-fluorouracil and leucovorin moderately improved overall survival in patients of metastatic pancreatic cancer (Wang-Gillam et al., 2016)]. Additionally, the features of the particle used to investigate the EPR effect will influence conclusions, and findings from laboratory animals are not necessarily transferable to the situation in humans (Figure 2).

In a comprehensive meta-analysis, the reported accumulation of nanoparticles in preclinical tumor models was analyzed and presented in terms of % injected dose (%ID) (Wilhelm et al., 2016). The published data (available from the paper's supplementary materials and the Cancer Nanomedicine Repository, http://inbs.med.utoronto.ca/CNR) also provides quantification of accumulation with a target quantity of % injected dose per g of tissue (%ID/g), which was investigated here to facilitate comparison with other studies. This revealed that the median accumulation, normalized to tissue mass, was highest for pancreatic tumors (5.8 %ID/g, range 1.8–13.4) and lowest for lung tumors (1.1, 0.04–45.8). This observation stands in apparent contradiction to the aforementioned hypovascular characteristic of pancreatic cancers. However, as outlined above, a multitude of other factors, such as particle characteristics or the type of tumor model also influences tendencies of accumulation. For example, in their original investigation, Wilhelm et al. conducted a multivariate analysis over their full dataset, in which *p*-values for the effect of particle diameter and tumor model on delivery efficiency (%ID) were not significant individually (>0.05), but the interaction of both terms was. These observations and trends illustrate the heterogeneity of the EPR effect even in rodent models, for which it is generally well-accepted.

**Figure 3:**
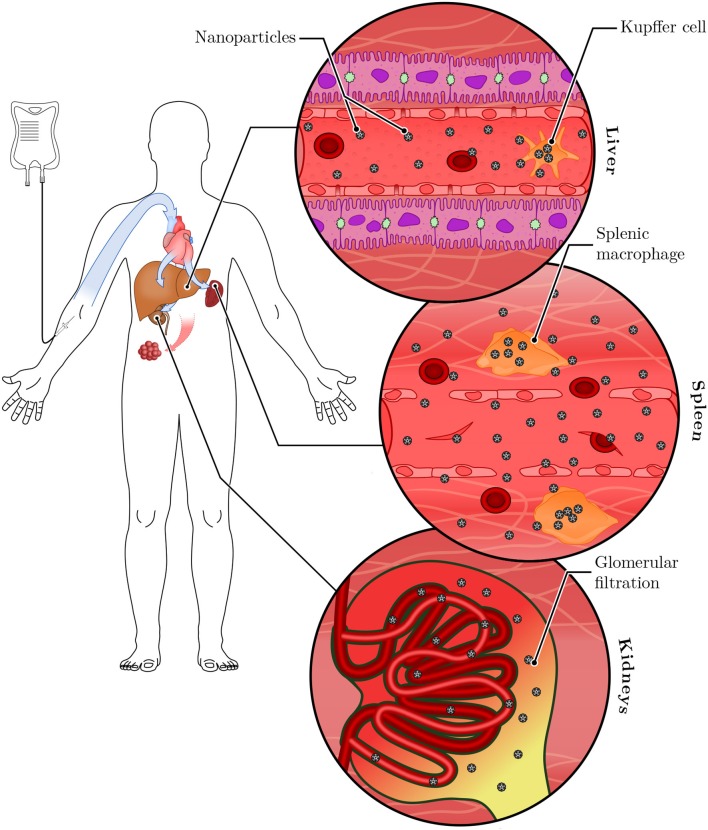
Systemic clearance of nanoparticles. Following intravenous injection, nanoparticles are distributed systemically through the bloodstream. They reach the liver and the spleen, where tissue-resident macrophages (called Kupffer cells in the liver) sequester a large portion of the administered dose. Nanoparticles small enough to pass the glomerular filter (below ~5 nm) are excreted in the urine. Remaining nanoparticles have the opportunity to accumulate in tumor tissues.

A large fraction of the injected dose is sequestered by tissue-resident macrophages before it can accumulate in the tumor tissue, and very small particles (below ~5 nm in diameter) may also be cleared in the kidneys (Figure 3). When the macrophage populations of the liver and spleen were depleted by pretreatment with clodronate liposomes, the fraction of particles found in the liver and spleen were reduced or increased, respectively (Tavares et al., 2017). Concomitantly, plasma half-life and tumor accumulation of gold nanoparticles both increased significantly. However, although a large relative increase compared to the non-depleted condition was found, absolute accumulation of particles in the tumor still did not exceed 2% ID, emphasizing that premature clearance by macrophages is not the only mechanism preventing efficient accumulation. Overall, the effects of macrophage depletion were found to be polymorphic for the different xenografted tumor models of human origin used by the authors: in an orthotopic MDA-MB-435S (melanoma) model, no increased tumor accumulation (in terms of % injected dose) was observed. In the orthotopic MDA-MB-231 (mammary adenocarcinoma), the heterotopic SKOV3 (ovarian adenocarcinoma) and the heterotopic A549 (lung carcinoma) models, a 20-fold increase was observed, whereas a 100-fold increase was achieved in the orthotopic PC3 (prostate carcinoma) model.

Small animal models are useful for characterization of the EPR effect and verifying the efficacy of new nanotherapeutic formulations, but the situation is even more complicated and inadequately understood for larger animals and humans. More recently, quantification attempts of long-term accumulation have been made. In a study with dogs,^64^Cu^2+^-labeled liposomes with a lipid composition equivalent to Doxil were used to quantitatively measure their deposition in various cancers by Positron Emission Tomography (PET) and Computed Tomography (CT) (Hansen et al., 2015). Liposome uptake in tumors increased from 1 to 24 h in 6 out of 7 carcinomas, but not in sarcomas. One of the dogs had metastases in the lung and axillary lymph node, in which liposome accumulation occurred. The achieved concentrations 24 h after administration ranged between 0.0048 and 0.0231 %ID/g for the carcinomas, and between 0.0011 and 0.0038 %ID/g for the sarcomas.

^64^Cu^2+^-based PET imaging was also used in a clinical study involving MM-302, a PEGylated liposomal formulation of doxorubicin with targeting activity toward HER2 (a growth factor receptor overexpressed in many mammary carcinomas) for the treatment of breast cancer (Lee et al., 2017). Tumor deposition was heterogeneous both within lesions of the same patient and between patients, and varied between ~0.001 and 0.01 %ID/g on day 2 after administration. A correlation between lesion size and carrier accumulation was not found. Deposition also occurred in normal liver, spleen and bone marrow, but not in other normal tissue, such as muscle.

Comparing the quantitative data of accumulation obtained from preclinical (rodent) studies (Wilhelm et al., 2016) and veterinary and human clinical observations (Hansen et al., 2015; Lee et al., 2017), a large discrepancy between both branches is evident, with rodent tumor models displaying a manifold higher uptake. This highlights the difficulty in translating observations from currently widely used investigative tools to the clinic due to profound differences in the underlying biological processes, such as the rate of growth or the size of malignancies relative to the host. Both of these are excessive in rodent models (Lammers et al., 2012a; Danhier, 2016). However, it has also been noted that focusing exclusively on the extent of accumulation omits other crucial parameters for the evaluation of drugs, such as their pharmacokinetic and toxicological properties, which can contribute to positive clinical outcomes, for instance by prolonging the exposure time to the compound (McNeil, 2016).

A big unmet need in cancer medicine is the effective extermination of metastases. In secondary lesions, the EPR effect will likely only be present in nodules that exceed the size threshold above which vascularization becomes a necessity, and will probably be equally heterogeneous. Although the physiological processes of abnormal vessel development underlying the EPR effect could be observed even in the initial phases of tumorigenesis (Hagendoorn et al., 2006), early and small lymphogenic metastases were not efficiently targeted by >150 nm liposomes (Mikada et al., 2017). However, 30 nm polymeric micelles loaded with a platinum complex suppressed lymph node metastases in a melanoma model, suggesting only a low grade EPR effect in these nodules (Cabral et al., 2015). In larger lesions, the EPR effect may eventually become significant, as exemplified by the efficiency of a pirarubicin-polymer against metastatic lung cancer (Tsukigawa et al., 2015) and the deposition of ^64^Cu^2+^-labeled liposomes in secondary lesions (Lee et al., 2017).

The specific tumor microenvironment affects extravasation of nanoparticles to a large extent, but the inverse may also be true. Recently, inorganic TiO_2_ nanoparticles were shown to promote gaps in between endothelial cells (Setyawati et al., 2017; Tay et al., 2017) and facilitate subsequent extravasation of cancer cells from the primary lesion to form metastases (Peng et al., 2019). Additionally, it has been suggested that liposomes not loaded with drug molecules may promote tumor growth and angiogenesis (Sabnani et al., 2015). Whether these are generalizable observations and whether they hold true for different types of inorganic or organic nanoparticles is presently unclear, but these questions are of potentially profound impact to the field of nanomedicine.

Thus, although the EPR effect is a reality in clinical settings, it is far from a simple manner and warrants critical evaluation (Figure 2). Exploiting it effectively remains a complicated challenge and will likely require individually tailored strategies in the clinic (Lammers et al., 2012b). Pharmaceutical interventions for enhancement of the EPR effect have been proposed (Fang et al., 2011), for instance by administration of hypertensive agents, such as angiotensin-II or by increasing vessel leakiness via NO-releasing compounds (such as nitroglycerine, which is used for the management of angina pectoris). On the other hand, inducing maturation of tumor vessels, for instance by inhibition of the VEGF signaling cascade, was reported to improve delivery of small and intermediately sized nanoparticles up to 40 nm to tumor tissue (Chauhan et al., 2012; Jiang et al., 2015) due to a reduction in the interstitial pressure and consequent dominance of convection over the less efficient diffusion (see next section). However, the effect on larger particles (above 60 nm) was minimal (Chauhan et al., 2012), because normalization reduced pore sizes and prevented extravasation of larger particles. Interestingly, in another report, normalization of highly aberrant vasculature by sorafenib (a kinase inhibitor, acting on the VEGF-induced signaling cascade) increased tumor retention of FITC-Dextran with a hydrodynamic diameter of ~50 nm. Conversely, the same inhibition in a tumor with vessels closer to the healthy state did reduce retention (Kano et al., 2009).

It has been suggested to use empty, non-drug loaded tracer particles, e.g., ^64^Cu^2+^-labeled liposomes, for estimation of the EPR effect for the individual patient in order to gauge potential therapeutic efficacy of a nanocarrier (Lee et al., 2018). However, this approach might in practice be hampered by the accelerated blood clearance (ABC) phenomenon, in which PEGylated nanocarriers are cleared more rapidly from the circulation upon repeated administration (Ishida and Kiwada, 2008; Abu Lila et al., 2013).

### 2.3. Distribution of Nanoparticles in the Tumor Mass

**Figure 4:**
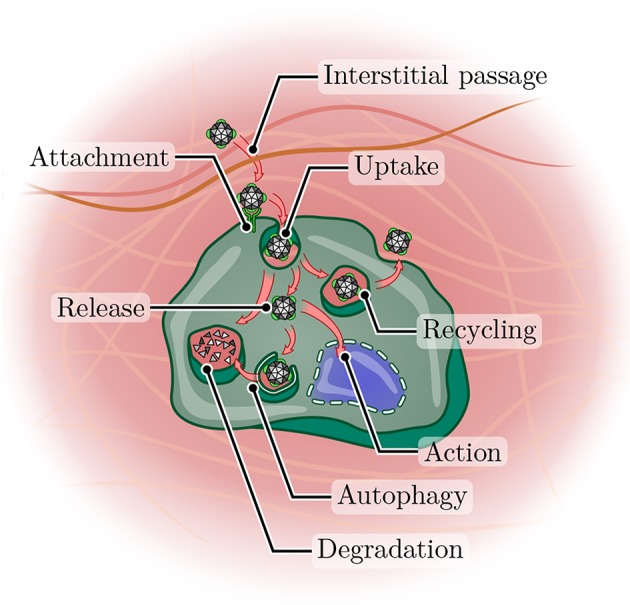
Journey of a nanoparticle toward its site of action. After arriving in a tumor tissue, a nanoparticle must first overcome the often dense interstitium before it can attach to the surface of a cancer cell (potentially with the aid of a targeting ligand). Uptake can occur via a variety of pathways (not shown), but frequently leads to arrival in the endosomal compartment. From here, the nanoparticle may enter the recycling pathway and undergo exocytosis, be routed toward lysosomal degradation or achieve release into the cytoplasm. Even then, autophagy may still lead to degradation, or the particle (or its cargo) may arrive at its site of action and perform its function.

After extravasation, the nanoparticles have not yet arrived at their final destination (Figure 4). They must be capable of maneuvering within the tumor mass to reach their target cells, a task made challenging by the difficult to navigate microenvironment of the tumor. One of the culprits is the high interstitial pressure in tumor tissue, to the elevation of which several factors contribute: Malignant cell proliferation results in an increase in cell mass, the overproduction of extracellular matrix components leads to fibrosis, and fluid pressure increases due to the leaky vasculature and the impaired lymphatic drainage (Chauhan et al., 2011). The increased interstitial pressure in tumor tissue diminishes the pressure gradient from circulation to tissue, especially in the central region of the lesion (Tong et al., 2004), where the lymphatics are more likely to be collapsed (Leu et al., 2000; Padera et al., 2002, 2004). This hampers convective transport processes and emphasizes undirected diffusion. Thus, all therapeutics achieve a slower net mass transport, contributing to their inefficient distribution in the tumor tissue, but this is especially problematic for nanoparticles due to their relatively larger sizes compared to free drug molecules, rendering their diffusion less efficient (Lane et al., 2015).

Centrally, the organization of extracellular matrix components, such as collagen or hyaluronic acid is also much more dense than in the periphery: Even within a few (1–2) millimeters from the surface of a murine B16 melanoma model, the diffusion capacity of macromolecules was substantially (up to several 100-fold) decreased compared to more superficial regions (Magzoub et al., 2007). This heterogeneity within one tumor is expanded by another layer of complexity in differences of diffusion for tumors of the same type, but at different localizations: When tumors derived from a glioblastoma or a melanoma were induced in mice, diffusion of macromolecules or liposomes was faster in tumors implanted at a cranial site compared to a dorsal site (Pluen et al., 2001).

Likewise, the distance that must be crossed also varies by tumor type (Smith et al., 2013): Based on the location of vessels, they were classified as “tumor vessels” (located close to cells) or “stromal vessels” (embedded within the stroma). For tumors rich in stromal vessels, diffusion distances to reach a target cell would be higher. This included colorectal, lung, prostate, and breast cancer model tumors, whereas vessels were closer to cells for renal, ovarian, hepatic, and thyroid cancers, as well as for a type of head and neck cancer and a glioma.

Recently, Zinger et al. proposed to utilize collagenase-loaded liposomes with slow release characteristics as a pretreatment to reduce the fibrotic character of pancreatic tumors and improve the delivery of secondary therapeutics (Zinger et al., 2019). They showed higher tumor weight reductions with the pretreatment and micellar paclitaxel, compared to micellar paclitaxel alone.

## 3. Shielding Strategies to Improve Circulation Half-Life

For a therapeutic nanoparticle to be of use *in vivo*, it has to circulate for a sufficiently long time to reach its target and fulfill its intended function. This is especially important for carriers intended to exploit the EPR effect, in light of its apparent discontinuous and inefficient nature (Matsumoto et al., 2016), in order to give the agent ample time for accumulation to occur. To confer long half-lives on nanoparticle formulations or proteins administered with a therapeutic intent, conjugation to polyethylene glycol (PEG) is by far the most common approach (Jokerst et al., 2011; Suk et al., 2016). The bulky, hydrophilic polymer increases the plasma half-life of modified particles by preventing excretion via the kidneys and blocking efficient uptake by phagocytic cells (Gref et al., 2000), in effect shielding them. The diminished cellular interaction is not exclusively due to steric hindrance. Upon exposure to biological fluids, nanoparticles acquire a new physicochemical identity due to the deposition of a dynamic layer of biomolecules on their surface, a process termed fouling which results in the establishment of a biocorona. It includes immunoglobulins, components of the complement system (Vu et al., 2019), coagulation factors and a plethora of other molecules, which fundamentally influences how cells perceive the coated particles (Giulimondi et al., 2019). It is now becoming increasingly appreciated that PEG alters the composition of the biocorona, which consequently influences cellular interactions and uptake modalities (Pelaz et al., 2015; Schöttler et al., 2016; Weiss et al., 2019). Notably, PEGylation is not a guaranteed way to raise biocompatibility, but its effect depends on the underlying material. For instance, PEGylation of two-dimensional graphene oxide nanosheets actually increased the cytokine levels secreted by challenged macrophages by facilitating diffusion and lodging within the cell membrane with subsequent activation of pro-inflammatory receptors (Luo et al., 2017).

### 3.1. Adverse Reactions to PEG

For many applications, PEG is generally considered biocompatible, safe and is successfully used clinically in many approved products (Webster et al., 2007, 2009; Jevševar et al., 2010). There is no doubt that PEG has made its mark on the market and is here to stay, however, it is not biologically inert and suffers from a number of issues. For instance, PEG is not biodegradable and has been shown to accumulate in the cytoplasm of renal cells, forming vacuoles whose impact on cell and organ function is unclear, but which may be of significance in the case of chronic applications (Bendele et al., 1998; Rudmann et al., 2013). Moreover, PEG may be more immunogenic than previously thought. Antibodies against PEG can be found after administration of PEGylated therapeutics, but also in more than 20% of naive healthy blood donors (Garay et al., 2012), as well as in naive pigs, with a potential source for priming being PEG contained in foods (Kozma et al., 2019). PEGylated asparaginase (PEG-ASNase) is used as a treatment for acute lymphoblastic leukemia. In a retrospective analysis of patient serum samples, the presence of anti-PEG was measured. In all of the samples in which anti-PEG could be detected, PEG-ASNase activity was undetectable, suggesting interference of these antibodies with the treatment (Armstrong et al., 2007). Considering the aforementioned potentially high prevalence of anti-PEG in the general population, such effects may be of considerable concern for the widespread use of PEG-modified nanocarriers. Apart from confounding treatment success, more immediately harmful effects were also seen: Hypersensitivity reactions were reported with PEG-containing therapeutics (Wenande and Garvey, 2016), both for PEG alone (used for instance for its laxative properties or as an excipient) or for bioconjugate products. Likewise, infusion reactions are prevalent for Doxil (Szebeni et al., 2002; Chanan-Khan et al., 2003). They occur in ~11% of patients and vary in their intensity, ranging from flushing to anaphylactic shocks (Janssen Products, 2019). However, not all of these events must necessarily be attributable to PEG, since infusion reactions also occur for un-PEGylated nanomedicines (Szebeni et al., 2018).

The immunological response against PEG appears to be responsible for the aforementioned ABC phenomenon, thus hampering individual evaluation of the EPR effect with empty liposomes on a per patient basis. The first administration of PEGylated nanocarriers causes a priming effect with induction of IgM antibodies which have an opsonizing activity for subsequent doses, resulting in activation of the complement system and rapid clearance (Abu Lila et al., 2013; Kozma et al., 2019). The ABC phenomenon appears to be less pronounced or absent in liposomes containing cytotoxic drugs, because rapid elimination of injected liposomes was only observed when their administration was preceded by another dose of empty PEGylated liposomes, but not of doxorubicin-loaded liposomes (Laverman et al., 2001). Toxic effects of the loaded drugs on macrophages (Laverman et al., 2001) or splenic B cells (Ishida et al., 2006) have been suggested to be responsible for this observation. Concerning the situation in human patients, in a small clinical study with 12 participants involving three cycles of Doxil treatment, no increasing clearance was observed (La-Beck et al., 2012).

### 3.2. Alternatives to PEG

There are multiple approaches under investigation to reduce PEG-associated immunity. For instance, it was shown that the incorporation of gangliosides (a type of glycolipids natively present in cell membranes) into PEGylated liposomes reduced production of anti-PEG IgM in challenged mice, but only when both ligands were presented on the same particle (Mima et al., 2017). When these ganglioside-containing PEGylated liposomes were administered as a pretreatment, they also reduced anti-IgM production upon subsequent treatment with PEGylated liposomes without gangliosides. This raises the potential for upgrading PEG-containing therapeutics—nevertheless, there is an extensive body of research on other shielding polymers.

Synthetic hydrophilic polymers used for the extension of circulation half-life include poly(N-[2-hydroxypropyl] methacrylamide) (HPMA), poly(vinylpyrrolidone) (PVP), poly(2methyl-2-oxazoline) (PMOX), poly(N-acryloyl morpholine) (PAcM), and poly(N,N-dimethylacrylamide) (PDMA). When the half-life extensions of liposomes grafted with these compounds were evaluated in a comparative study, PMOX and PEG were found to have the largest effect (Kierstead et al., 2015). However, the authors note that an optimization of chain length and grafting had not been performed for polymers other than PEG, suggesting their performance may be improved. Notably, shielding efficiency was correlated to polymer viscosity, and the two most viscous polymers (PEG and PMOX) were the only ones to induce the ABC phenomenon in this study. However, a causality of this observation was not shown, and it is thus only based on correlation.

A promising biological alternative to PEG is PAS: PAS is a heteropolymeric amino acid sequence incorporating the three monomers proline, alanine and serine (Schlapschy et al., 2013). These amino acids adopt an unfolded disordered conformation if assembled in a suitable manner, thus exposing the hydrophilic polypeptide backbone (with only a minor contribution of the serine hydroxy group to the overall hydrophilicity) (Breibeck and Skerra, 2018). One PAS unit consists of ~20 monomers, and multiple PAS repeats can be chained to increase the shielding effect. The resulting polymeric sequence has biophysical properties which are similar to those of PEG, conferring an increased hydrodynamic radius on its fusion partners. As with PEG, this property varies with chain length, and a 400 residue PAS sequence is approximately equivalent to a 30 kDa linear PEG chain in its elution characteristics from a size exclusion matrix (Breibeck and Skerra, 2018).

Because PAS is an amino acid sequence, it can be genetically encoded. This obviates the need for post-translational chemical conjugation of therapeutic proteins, which carries the disadvantageous potential to alter their activity. Moreover, whereas preparations of PEG are polydisperse due to their synthesis, PAS is produced by the highly precise cellular machinery, reducing heterogeneity. From an economical point of view, PASylation likewise offers attractive prospects: Expensive raw materials are not necessary, and additional purification steps after conjugation, which reduce yields and are costly in themselves, are not required.

PAS has been used *in vivo* to extend the circulation half-life of proteins, for example of somatotropin (human growth hormone, hGH) (Schlapschy et al., 2013), antibody fragments (Mendler et al., 2015), leptins (Morath et al., 2015; Bolze et al., 2016), and interferon (Xia et al., 2019). For the hGH-PAS fusion protein, the immunogenicity of PAS was investigated—while antibodies against hGH-PAS did arise, epitope mapping revealed these were not reactive toward the PAS chain, but against epitopes of hGH, and no cross-reactivity to other PASylated proteins was found. However, given that antibody responses against multimeric epitopes are often strong due to their structural similarity to viral capsids (Bachmann and Zinkernagel, 1996; Yankai et al., 2006; Ogun et al., 2008), and in light of the inherent limitations of animal models for the investigation of immunogenicity, it is conceivable that antibodies against PAS or other immunological phenomena may be found if it is used in clinical practice. Nevertheless, data so far indicated that PAS was well-metabolizable and did not result in histological irregularities in the liver, spleen or kidneys of mice (Schlapschy et al., 2013).

PAS may also be an option for modification of nanoparticulate formulations other than therapeutic proteins. A protein nanocage composed of the ferritin heavy chain (HFt), utilized for the entrapment of doxorubicin, was PASylated by fusing its constituent monomers to 40 or 75 residue PAS sequences (Falvo et al., 2016). The PASylated variants showed chain-length dependent extended plasma half-lives of the entrapped doxorubicin in intravenously injected mice.

Polysarcosine (PSar) is a polymer of sarcosine (N-methylated glycine, an intermediate of glycine metabolism) in which tertiary amide bonds in the backbone confer resistance to proteolytic degradation compared to conventional peptides (Miller et al., 1995). Similar to PEG, PSar could be conjugated to lipids, potentially allowing the generation of PSar-protected liposomes and micelles (Weber et al., 2016). When PSar was grafted on a TiO_2_ sheet, it reduced the amount of fibrinogen associated with the surface and prevented the attachment of mammalian and bacterial cells (Lau et al., 2012). In another study (Hu et al., 2018), interferon 2β was modified either with a 12 kDa PSar chain, or a 10 kDa PEG chain, which resulted in similar hydrodynamic radii and almost identical plasma half-lives. Accumulation in tumors of a murine OVCAR3 (ovarian carcinoma) model was slightly higher for the PSar-modified variant, culminating in an increased inhibition of tumor growth after 25 days, whereas liver deposition was lower. Data also suggested that less anti-IFN IgG was produced in response to PSar-IFN, but antibodies against PSar proper (i.e., antibodies binding directly to PSar) were not assessed. A block-copolymer consisting of poly(L-lactic acid) and PSar (lactosome) showed accumulation in murine tumor models of orthotopic hepatic and lung carcinomas, and a heterotopic pancreas carcinoma (Makino et al., 2009). However, upon repeated administration, lactosome was prone to an ABC phenomenon and was cleared rapidly from the circulation by hepatic sequestration (Hara et al., 2012). This metabolic clearance correlated with production of IgM and IgG3 which bound to PSar and persisted for 6 months after the first administration. Furthermore, the immunogenicity of PSar was found to vary with the type of particle (polymeric micelles or vesicular), the hydrodynamic diameter and the membrane elasticity (Kim et al., 2017).

An entirely different strategy to confer the ability to avoid rapid clearance on nanoparticles relies on the exploitation of the naturally occurring membrane composition of red blood cells (RBCs): RBCs were lysed, their membrane fraction was collected and subsequently grafted onto poly(lactic-co-glycolic acid) (PLGA) nanoparticles by passing the membrane suspension together with the particles through an extrusion device with a pore size of 100 nm. Following this treatment, the nanoparticles were covered in the RBC membrane and displayed increased *in vivo* half-life compared to conventionally PEGylated PLGA nanoparticles (Hu et al., 2011). In a subsequent study, the authors showed that this property was to some extent dependent on the presence of CD47 on the membrane surface, a glycosylated transmembrane protein which acts as a marker of “self” for the immune system and reduces uptake of the covered nanoparticles by macrophages due to binding SIRP (signal-regulatory protein) (Brown and Frazier, 2001). The increased half-life was not entirely abrogated upon blockade of CD47 by antibodies, however, suggesting involvement of other components which act as a shielding layer, e.g., the RBC glycocalyx, which was grafted in concert (Hu et al., 2013). In a different setup, adding an additional shielding layer by genetically engineering HEK293 (human embryonic kidney) cells to express PAS on their surface and grafting their membrane onto PLGA nanoparticles further increased the *in vivo* half-life in comparison to cell membranes without PAS (Krishnamurthy et al., 2019). For human use, such an approach naturally begs the question of how to handle immune reactivity from different blood groups or potential contamination by pathogens. One option may be the use of autologous erythrocytes for personalized nanoparticle coatings.

### 3.3. The PEG Dilemma and Stimulus-Responsive Release

Irrespective of the strategy chosen for the avoidance of renal clearance and the MPS, there is a disadvantage associated with the prevention of cellular interactions: After successfully evading phagocytosis along the way and having arrived at its destined site of action, for instance by virtue of the EPR effect, the nanoparticle in question is now incapable of efficiently interacting with its target cells, such as tumor cells (Mishra et al., 2004). Furthermore, their efficient endosomal escape is impeded, resulting instead in the delivery of the particles into the degradative lysosomal pathway (Remaut et al., 2007; Dominska and Dykxhoorn, 2010).

As such, different strategies have been developed to allow nanoparticles to shed their protective coat or release their cargo in order to facilitate efficient uptake by their target cells (Hatakeyama et al., 2011; Zhu and Torchilin, 2013). For this purpose, nanoparticles have been engineered with the ability to change their properties in a stimulus-dependent fashion. Broadly speaking, such approaches can be divided into two distinct categories: Intrinsic and extrinsic stimulus-responsiveness (Mura et al., 2013; Jin et al., 2019).

Intrinsic stimulation relies on locally variable environmental circumstances (for instance in the tumor microenvironment), such as pH, redox potential or enzymatic activity. Contrarily, extrinsic stimulus-responsive nanocarriers are intended to react to cues applied externally by the treating physician at a precisely defined location and/or time, for instance by exposure to light, heat, ultrasonication or a magnetic field. Combinatorial approaches make for a monumental number of possible systems. A few recent illustrative examples highlighting the breadth of potential mechanisms are given below; For a more thorough overview, the interested reader is referred to other excellent reviews on this topic (Mura et al., 2013; Wang and Kohane, 2017; El-Sawy et al., 2018; Jin et al., 2019).

Nanocarriers may react to stimulation, whether extrinsic or intrinsic, either by the release of the free, low molecular weight drug, by shedding of the shielding layer (allowing facilitated uptake of the drug-loaded nanocarrier), or by altering their properties, such as size or geometry. Variants which keep the nanocarrier intact but facilitate its uptake, and therefore enable delivery of a bulk payload to target cells, bear the potential of overcoming drug resistance in resilient malignancies by three mechanisms: First, nanoparticles can deliver their payload via the endocytic internalization pathway, thus bypassing drug efflux pumps, such as P-glycoprotein (P-gp), which are localized in the plasma membrane, or exceeding their capacity after release of the active compound from the endosomal system (Davis et al., 2008; Wang et al., 2014). Second, by loading nanocarriers with synergistic combinations of compounds, therapeutic efficacy can be higher than for the individual drugs, and the evolution of drug resistance can be slowed down or prevented (Parhi et al., 2012). This is the principle behind Vyxeos, a liposomally encapsulated combination of cytarabine and daunorubicin (Lancet et al., 2018). Third, inhibitors of P-gp can be co-loaded with the therapeutic drug and delivered in concert (Saneja et al., 2014), for example chemical inhibitors (Tang et al., 2016) or siRNA (Zhang et al., 2016).

#### 3.3.1. Intrinsic Stimulation

Matrix-metalloproteinases (MMPs) are a family of proteases involved in the progression of cancer, for instance by releasing growth factors from the extracellular matrix, mediating angiogenic processes, or facilitating invasive and migratory phenotypes by carving a path through the matrix (Kessenbrock et al., 2010). By conjugating PEG to a peptide comprising the recognition sequence of MMP-2, the PEG layer was rendered cleavable in the presence of this protease. The conjugate was then coupled to a cholesterol anchor and inserted into the lipid bilayer of liposomes by post-insertion (Wan et al., 2013). Encapsulating adenoviral vectors in these liposomes allowed higher transduction efficiencies of an MMP-2 secreting tumor cell line when cleavable PEG was grafted on the liposomes, compared to non-cleavable PEG.

Due to oxidative stress in the tumor area, increased amounts of the reducing tripeptide glutathione have been reported there. To exploit this phenomenon, a reduction-sensitive PEGylated lipid (1-palmitoyl-2-oleoyl-sn-glycero-3-phosphoethanolamine, POPE), linking PEG to a glycerophospholipid via a disulfide bridge (POPE-SS-PEG5000) was synthesized. As a second stimulus-responsive component, a lipopeptide mimicking the triple-helical structure of collagen which was cleavable by MMP-9 was incorporated into liposomes (Kulkarni et al., 2014). Exposure of these liposomes to glutathione resulted in the shedding of the PEG layer, subsequently providing access to the MMP-9 cleavage site of the lipopeptide, which destabilized the triple helix and allowed release of liposomally encapsulated contents. Notably, the destabilization process increased the size of the vesicles, possibly owing to aggregation resulting from the destabilization. In a heterotopic murine model of the human pancreatic cancer cell line PANC-1, these double-responsive liposomes showed preferential release of carboxyfluorescein at the tumor site. When liposomes were loaded with gemcitabine, treatment with vesicles containing the lipopeptide resulted in a moderately larger reduction of tumor growth compared to liposomes not responsive to MMP-9.

The pH in malignant lesions is typically lowered compared to healthy tissues because of their heightened metabolic activity, allowing potential discrimination of the tissue disease state (Lee et al., 2008). Additionally, the intracellular space is reducing, in contrast to the oxidizing extracellular compartment. One example of a pH and redox dual responsive nanocarrier is the iCluster system developed by Li et al. (2016). They linked polycaprolactone (PCL) and polyamidoamine (PAMAM) with a pH-sensitive linker (2-propionic-3-methylmaleic anhydride; CDM). The resulting PCL-CDM-PAMAM polymer was conjugated to a platinum prodrug and co-assembled with a PEG-PCL heteropolymer. The complete nanocarriers were ~100 nm in size and exhibited a long plasma half-life by virtue of the incorporated PEG. Upon encounter of a slightly acidic milieu (pH 6.8), the CDM-based linker was cleaved, releasing substantially smaller PAMAM/Pt particles about 5 nm in size. The rationale behind this design was to facilitate diffusion through the tumor interstitium by size-reduction after accumulation at the site of interest via the EPR effect. The small particles were then taken up by cells and, upon encounter of the reducing conditions of the intracellular space, cisplatin was released from PAMAM/Pt, resulting in cytotoxicity. In murine models of heterotopic xenografts of Bx-PC3 human pancreatic tumor and a cisplatin-resistant A549R human lung tumor, the pH- and redox-sensitive cluster showed higher tumor growth inhibition and prolongation of survival compared to free cisplatin, PAMAM/Pt or a pH-insensitive cluster variant. An extension of median survival was also reported for an orthotopic allograft of the metastatic 4T1 mammary carcinoma.

A form of stimulus-responsiveness may also be involved in the mechanism of action of Doxil. Doxorubicin is remotely loaded into liposomes by means of an ammonium sulfate gradient, which allows the uncharged, unprotonated doxorubicin to diffuse through the membrane into the liposomal interior space, where a high ammonium sulfate concentration and low pH cause it to be protonated and form crystalline rods with the abundant sulfate ions (Haran et al., 1993; Wei et al., 2018). Extensive glutaminolysis at the tumor site could produce NH_3_, which diffuses through the liposomal membrane, receives a proton from doxorubicin-${\text{NH}}_{3}^{+}$ and allows the now uncharged doxorubicin to diffuse out of the liposome again (Silverman and Barenholz, 2015).

For passively stimulated nanoparticles, no exogenous input is required, and consequently, no information about the localization of a lesion is necessary to induce release. However, this could simultaneously be considered a blessing and a curse, since precise yet uncontrollable perturbations are required for the systems' functionality. As outlined above for the EPR effect, such tumor-related phenomena can be highly heterogeneous (Marusyk and Polyak, 2010; Alizadeh et al., 2015).

#### 3.3.2. Extrinsic Stimulation

Conversely, active triggering does rely on external application of a stimulus. This requires knowledge about the target's location and extent, which renders this approach problematic for the eradication of very small and dispersed metastatic foci, but offers promising perspectives for the treatment of localized sites.

After allowing sufficient time for accumulation at the tumor site, suitably designed nanocarriers can be externally stimulated to induce efficient cargo release or allow cellular uptake. Light is a highly attractive modality for targeted activation due to the high spatiotemporal resolution of the stimulus. UV light is sufficiently energetic to cleave chemical bonds. However, for light-regulated systems, low-energy light in the far-red or near-infrared (NIR) region of the spectrum is most desirable for biological applications because it exhibits the highest capacity to penetrate tissues and is less cytotoxic compared to light of shorter wavelengths. For applications for which a high input of energy is nonetheless desirable, upconversion nanoparticles (UCNPs) offer tempting possibilities by virtue of an *anti-Stokes shift*, which converts multiple low energy input photons to higher energy output photons (Wen et al., 2018).

Photodynamic therapy (PDT) is a treatment modality which relies on the generation of cytotoxic reactive oxygen species (ROS) by means of photosensitizers after light illumination (Lucky et al., 2015). PDT is clinically tested for the treatment of malignancies for example of the skin, bladder, or esophagus, which are situated in proximity to an interior or exterior body lining (and therefore allow external illumination), but is of limited efficacy when the tumor harbors hypoxic regions. Conjugating photosensitizers to nanoparticles can improve their pharmacokinetic profile and bestow additional functionalities upon them. In an intricate setup (Xu et al., 2018), micelles of CPP (which is the PEG-modified photosensitizer chlorin e6 conjugated to a non-cytotoxic platinum(IV) diazido complex) were associated with UCNPs, which can convert inbound 980 nm light to emissions of 365 and 660 nm. Illumination with 980 nm light lead to formation of cytotoxic Pt(II) and O_2_, allowing the generation of ROS even in the hypoxic tumor environment. The nanoparticles showed accumulation in the tumor and high anti-tumor activity in murine models of four different tumor cell lines upon illumination.

Shedding of PEG by NIR light was achieved by utilizing a UV-sensitive *o*-nitrobenzyl (Nbz) linker, connecting PEG to poly-β-aminoesters (PAE; PEG-Nbz-PAE-NBz-PEG). By incorporation of UCNPs, this setup allowed release of encapsulated doxorubicin in response to NIR illumination, which first led to removal of the PEG layer by the upconverted UV irradiation and also allowed cleavage of the pH sensitive PAE (Zhou et al., 2019).

To confer thermosensitivity on doxorubicin-loaded liposomes, a formulation that incorporated lyso-lipids into the phospholipid bilayer was developed (lyso-thermosensitive liposomes, LTSL; marketed as ThermoDox), which lowered the phase transition temperature and allowed rapid drug release (within 20 s) at moderately elevated temperatures of 39–40°C (Needham et al., 2000). Heating *in situ* can be achieved as a side-effect of radiofrequency ablation (RFA), which is based on the application of an alternating current to the tumor via an inserted probe. The invasiveness of this procedure depends on the route of access. In a clinical phase 3 study, LTSL were injected systemically to patients with unresectable hepatocellular carcinoma, and local release was induced by heating via RFA (Tak et al., 2018). An initial analysis did not find differences for overall or progression-free survival between patients treated with RFA and placebo, or with RFA and LTSL in combination. However, the original treatment protocol did not specify the exact duration of RFA treatment. When the analysis was restricted to patients who underwent RFA for more than 45 min, a significant improvement in overall survival was found for the RFA + LTSL arm. Since this analysis was performed *post-hoc* for hypothesis generation, a follow-up prospective study was planned and performed (NCT02112656), but results have not yet been made available.

In a phase 1 study with 10 patients suffering from primary or secondary liver tumors, the stimulus for heating in order to release doxorubicin from LTSL was applied externally by high-intensity focused ultrasound (HIFU), without the need for invasive placement of a probe, although the first part of the study employed an implanted thermosensor to allow tuning of the ultrasound parameters to appropriate levels (Lyon et al., 2018). Biopsies of tumor tissue were taken after infusion of LTSL, and again after application of the ultrasound pulse. After application of ultrasound, doxorubicin levels were on average 3.3 times higher than before, and doxorubicin fluorescence co-localized with the nucleus in tissue sections of the HIFU-treated biopsies. Tumor sizes were monitored by PET-CT after the intervention. In some patients, tumors which had not been targeted by HIFU were visible on the same imaging plane as targeted tumors. Crucially, the targeted lesions shrank more substantially compared to the untargeted lesions, demonstrating the efficiency of the stimulation approach in a clinical setting with visible benefits.

## 4. Targeting

For cancer nanomedicine, directing severely toxic drugs to their site of action is a goal of utmost importance. Partially, this is achievable as a consequence of the EPR effect, however, as outlined above, relying exclusively on this phenomenon may be insufficient, and more advanced approaches are under investigation.

Employing magnetism to influence suitably responsive particles carries great potential for non-invasive interventions (Prijic and Sersa, 2011; Tietze et al., 2015). Of these, mainly superparamagnetic iron oxide nanoparticles (SPIONs) are investigated for diagnostic or therapeutic purposes, for instance as contrast agents for magnetic resonance imaging or to induce local heating for hyperthermic ablation of tumor cells or other thermoresponsive systems. Additionally, their unique properties allow them to be locally targeted by the application of an external magnetic field. After promising preclinical results, this magnetic drug targeting had entered clinical trials for hepatocellular carcinoma in the form of MTC-Dox (magnetically targeted carriers with doxorubicin), particles of about 0.5–5 μm in size (hence not strictly meeting classification as nanoparticles) and composed of iron and carbon to which doxorubicin was adsorbed passively (Rudge et al., 2001). However, a therapeutic trial was terminated due to not achieving preset endpoints (NCT00034333). Notably, to prevent clearance and systemic distribution, these studies involved administration of the particles via an intraarterial catheter of the tumor-feeding artery, where extravasation was then induced by placing a magnet above the abdominal wall.

Magnetic targeting continues to be explored for its potential to achieve high local drug loads. For instance, a study with rabbits found over 50% of the applied drug load in the tumor tissue after intraarterial administration and magnetic targeting of 200 nm lauric acid coated SPIONs loaded with mitoxantrone to a superficial tumor grafted in the hind legs (Tietze et al., 2013). Al-Jamal et al. used PEGylated (thus longer circulating) nanoparticles with oil cores and varying amounts of incorporated SPIONs to quantitatively study magnetic particle targeting after intravenous injection (Al-Jamal et al., 2016). Extrapolating their mathematical model from murine data to potential human use, they suggest magnetic targeting under their conditions to be sufficient to achieve targeting in clinical practice.

A hitherto unsolved challenge is the magnetic targeting of deep tissues, due to issues with focusing magnetic fields and rapidly decaying magnetic field strength with distance (Shapiro et al., 2015). As such, the majority of studies conducted so far have focused on superficial tumors, which are easily reached by placing a magnet adjacent to the lesion.

One more dimension is added by kinetic targeting, which takes exploitation of the EPR effect a step further: In a pilot study involving 12 breast cancer patients and 3 ovarian cancer patients receiving PEGylated liposomal doxorubicin (PLD), extracorporeal plasmapheresis was applied 42–48 h after PLD treatment to remove residual circulating liposomes (Eckes et al., 2011). The treatment substantially reduced the doxorubicin plasma AUC by 50% and helped to alleviate undesirable side effects, such as palmar-plantar erythrodysesthesia (PPE, or hand-foot syndrome). PPE is a typical adverse reaction to PLD due to its pharmacokinetic profile, which allows accumulation of the drug in the skin especially of the hands and feet, where lesions can occur, but this accumulation is slower in skin compared to tumors (Charrois and Allen, 2003). PPE can be dose-limiting in some instances. During a total of 57 cycles of PLD and plasmapheresis, PPE occurred only in a single patient, whereas previous comparable trials without plasmapheresis reported occurrence in a total of 8/33 patients. No occurrence of grade IV neutropenia was reported (previous studies: 8/33). These data suggest an improvement of the toxicological profile of PLD and possibly other nanosized drug formulations by reducing systemic exposure to these agents via plasmapheresis. The response rate to the treatment appeared to be comparable to previously conducted studies, but in this pilot study, no comparative arm without plasmapheresis was included.

In addition to the approaches described here, which aim at improving the accumulation of particles in the tumor *tissue*, the next step is to target particles to individual tumor *cells* to improve uptake and cargo delivery.

### 4.1. Targeting Nanocarriers to Cancer Cells

Achieving cell-specific delivery is pursued by grafting targeting molecules onto the surface of nanoparticles. Many different monoclonal antibodies binding to cell surface molecules upregulated on cancer cells are now in clinical use, and similar approaches have been exploited for use in nanomedicine. MM-302 was briefly described above: It is a PEGylated liposomal formulation of doxorubicin to which a single chain variable fragment (scFv) against HER2 is bound via PEG as a spacer (Miller et al., 2016). ScFvs are synthetic proteins of the variable regions of an antibody molecule connected by a short peptide linker. Importantly, they lack the Fc region which interacts with Fc receptors of many immune cell populations, and only harbor the targeting activity. Other targeting ligands also exploit the overexpression of cancer antigens, such as the folate receptor (folic acid conjugated particles, e.g., Lu et al., 2012; Tang et al., 2018) or the transferrin receptor (transferrin conjugated particles, e.g., Sarisozen et al., 2014; Wei et al., 2019). Transferrin is also under investigation as a ligand to induce transcytosis across the blood brain barrier, thus enabling cerebral delivery of nanotherapeutics (Ulbrich et al., 2009; Wiley et al., 2013; Clark and Davis, 2015). Furthermore, interaction between complementary DNA strands on liposomes and target cells were shown to improve delivery of the nanoparticles (Li et al., 2019), and grafting single-stranded DNA to antibodies facilitated their intracellular delivery in a sequence-independent fashion (Herrmann et al., 2019).

Alternatively, grafting complete cancer cell membrane fractions onto nanoparticles to allow homotypic interaction with the tumor cells by virtue of their adhesion molecules may be possible (Fang et al., 2014). This might enable more strictly personalized targeting, however, since a membrane source is required for such an approach, it would likely be reserved for accessible tumors in order to allow gathering sufficient material.

Although many preclinical studies report tremendously encouraging results using targeted nanoparticles, existing data also suggests heterogeneous efficacy of ligand-mediated targeting, where the targeting ligand was capable of improving cellular uptake and altering the uptake pathway (Pirollo and Chang, 2008; Clemons et al., 2018). However, localization of the particle to the tumor tissue was not necessarily improved, depending on particle size and the presence of PEG, with PEG potentially masking accumulation effects because of exploitation of the EPR effect (Pirollo and Chang, 2008; Choi et al., 2010). Moreover, caution should be exercised when extrapolating from *in vitro* to *in vivo* data, since the establishment of a biocorona can substantially impact targeting abilities (Salvati et al., 2013; Francia et al., 2019). The abrogation of targeting by serum protein binding can be alleviated by additionally grafting PEG, which is of lower molecular weight or length used to conjugate the targeting ligand to the particle surface. The polymer may then help to reduce fouling while not sterically hindering binding to the target molecule (Dai et al., 2014).

More recently, by using very small HER2-functionalized silica nanoparticles (~7 nm), tumor targeting efficiencies of 10.3–17.2% ID/g were achieved in murine xenografts of HER2-positive BT-474 tumors, compared to 3.3–6.1% ID/g for HER2-negative tumors or untargeted particles (Chen et al., 2018). However, their fate after accumulation in the tumor was not further followed.

To address this question, Dai et al. quantitatively investigated the effects of active targeting (Dai et al., 2018). Using gold nanoparticles conjugated to trastuzumab (a monoclonal antibody against HER2), they used different particle sizes and tumor models to measure particle distribution in tumor tissue. For an ovarian SKOV-3 cancer model, xenografted subcutaneously to murine hosts, 0.59% of the injected targeted particle dose reached the tumor, whereas this amounted to 0.25% of untargeted particles. These values were in line with a previous meta-analysis of preclinical models published by the group earlier, where the median accumulation of actively targeted particles was 0.9% ID, versus 0.6% ID for passively targeted particles (Wilhelm et al., 2016). Strikingly, they found that even for particles that reached the tumor site, these were much more likely to be stuck in the acellular matrix (typically over 90% of all particles) of the tumor, or to be engulfed by tumor-associated macrophages (TAMs), than to be taken up by cancer cells. Furthermore, the difference in the fraction of particles taken up by cancer cells was not statistically significant between targeted and untargeted nanoparticles (0.001 vs. 0.003% ID for targeted and untargeted, respectively), and neither was the difference in the fraction of tumor cells that engulfed particles (0.96 vs. 0.42% for targeted and untargeted, respectively). These results were corroborated by follow-up experiments using other tumor models. When nanoparticles which were targeted to folate instead of HER2 were investigated, TAMs still dominantly engulfed nanoparticles. The authors suggest that this observation is in line with the perivascular localization of TAMs, making them more likely to first capture incoming nanoparticles, thus acting as a filter before malignant cells have an opportunity for interaction.

Moreover, targeting may not always be favorable, depending on the carrier: When small polymeric nanoparticles (10 nm) were actively targeted to tumor endothelium by grafting of RGD- or NGR-peptides, they were found to accumulate much more strongly in the tumor tissue than their non-targeted counter parts at early time points, up to 4 h after injection, before the EPR effect became significant. However, for later time points (24–72 h), this trend was reversed, and the passively targeted carriers actually accumulated to a higher degree over time (Kunjachan et al., 2014). In this instance, the targeted carriers were also more likely to be found at off-target sites.

Since targeting moieties attached to a nanoparticle's surface typically induce receptor-mediated endocytosis, the next stage of the journey is the cellular vesicular system.

### 4.2. Escaping the Endosome and the Vesicular System

In Kafka's novel “The Trial,” the protagonist Josef K. is accused of committing an elusive crime, and subsequently desperately tries to navigate the intangible and confusing labyrinth of a convoluted court without ever reaching the higher tiers. It is a fate not quite unlike that of a nanocarrier, which, after finally reaching its target cell, has still not arrived at its ultimate goal.

Intact nanoparticles are principally believed to enter cells via the endocytic pathway, i.e., by attachment to the cell surface and subsequent incorporation into an intracellularly trafficked vesicle (Sahay et al., 2010; Zhang et al., 2015) (Figure 4). Roughly, endocytosis is divisible into two main branches (Dobrovolskaia and McNeil, 2007; Doherty and McMahon, 2009): Phagocytosis, which is the uptake of large particles or pathogens by specialized immune cells, and pinocytosis, which is the uptake of smaller particles and includes macropinocytosis and clathrin- or caveolin-dependent endocytosis.

Nanoparticle uptake depends on the dynamics of endocytosis, and on the dynamics of the attachment of particles to the cell surface. When nanoparticle uptake was investigated in two human cell lines using 8 nm quantum dots, the average number of nanoparticles found in a single endosome remained relatively constant with increasing concentration, however, the number of particle-containing endosomes was positively correlated with dose, at least in the range of doses and exposure times investigated (0.5–5 nM, and 0.5–2 h, respectively) (Rees et al., 2019).

Membrane scission of budding endocytic vesicle is typically induced by a family of dynamin GTPases. The primary vesicles then continue to fuse with early endosomes, which are the primary sorting station of the endosomal system and bear the potential to exclude a large fraction of incoming cargo by rerouting them toward exocytosis (Huotari and Helenius, 2011). Early endosomes mature to become late endosomes, with a concomitant drop in pH by the action of V-type ATPases, and eventually fuse with lysosomes, forming endolysosomes where enzymatic degradation of a variety of cargoes, such as nucleic acids, lipids and proteins occurs. The importance of these routes varies for different nanoparticle formulations: Stimulus-responsive systems which culminate in release of the free drug molecules at the disease site do not require endosomal escape, if the drug can diffuse across membrane barriers. However, for example for delivery of siRNA or DNA, the carrier has to be engulfed whole and needs a way to deliver its payload to the cytosol to prevent lysosomal degradation.

As briefly mentioned above, a large fraction of incoming cargo never proceeds very far into the endosomal system. For instance, for lipid nanoparticles (LNPs) intended for the delivery of siRNA, it was found that they entered cells via macropinocytosis, but about 70% of them were recycled and exocytosed by a mechanism involving the Niemann-Pick type C1 (NPC1) protein, which is a protein linked to a form of lysosomal storage disease (Sahay et al., 2013). Another study underscores that endosomal escape is an inefficient process: When siRNA-carrying lipid nanoparticles were traced, <2% were successful in reaching the cytosol (Gilleron et al., 2013).

Delivery of nucleic acids is often achieved by utilizing cationic polymers or lipids, such as poly(ethylene imine), which form complexes with the negatively charged phosphate backbone. The resulting polyplexes are believed to escape the endosome by exerting a “proton sponge” effect, in which the basic polymers buffer the proton influx during the acidification process, leading to a subsequent influx of chloride ions with a consequent endosomal swelling and rupture. However, this proposed mechanism is not undisputed and destabilization of the vesicular membrane by interaction with the polymer was proposed to induce endosomal leakage (Bus et al., 2018; Vermeulen et al., 2018).

Cationic lipid-based vesicles were more prone to aggregate in serum compared to their neutral counterparts, resulting in reduced tumor penetration and toxic side effects (Fischer et al., 2003; Zhao et al., 2011). Many approaches have been pursued to upgrade other materials with the capacity to effectively penetrate the endosome by swelling, membrane disruption, or potentially the proton sponge effect (Cupic et al., 2018; Smith et al., 2019). Many of these strategies rely on disruption of membrane integrity when the pH is lowered below physiological levels of the extracellular space, which might be hampered in the case of cancer nanomedicine due to the aforementioned lowered extracellular pH.

Even after successful endosomal escape, autophagy may pose a potential additional barrier. Autophagy (or, more precisely, macroautophagy, to distinguish this process from other forms of autophagy) is a cellular self-digestion mechanism which is induced upon starvation conditions to mobilize carbohydrates, lipids and proteins, but also contributes to the removal of damaged organelles (Levine et al., 2011). It involves the generation of a double isolation membrane (the phagophore) which encloses putative cargo and seals to form the autophagosome, which then fuses with lysosomes. In the resulting autolysosome, the vesicular contents are degraded. Cationic gene delivery polyplexes and liposomes were shown to be captured in autophagosomes, and in cells deficient for *atg5* (an essential regulator of autophagy), gene delivery was increased by a factor of eight (Roberts et al., 2013). Nanoparticles could also be captured by autophagosomes when they entered the cells via microinjection, bypassing the endosomal system, which suggests that once-escaped carriers may be removed from the cytosol again, limiting their time to perform a biological function (Remaut et al., 2014). Inhibition of autophagy by chloroquine was also reported to result in slower tumor growth of docetaxel-loaded PLGA nanocarriers (Zhang et al., 2014). However, because autophagy inhibitors are also investigated as cancer therapeutics (Kimura et al., 2013), it is unclear whether this was a result of a synergistic effect between the compounds leading to more efficient retention of the nanocarriers, or simply an enhancement by employing combination therapy. On the other hand, no major influence of *atg5* on knockdown efficiency was seen in a study involving LNPs and siRNA (Sahay et al., 2013).

### 4.3. Entering the Nucleus

Contrary to siRNA or mRNA, which can perform their function in the cytosol, DNA has to enter the nucleus to be transcribed. For the delivery of DNA, there are currently no approved non-viral vectors available, although clinical trials are in progress and intense research is underway, because synthetic alternatives promise advantages, such as reduced immunogenicity and oncogenic potential, as well as increased packaging capacity (Yin et al., 2014). Delivery of coding DNA is an attractive prospect for the generation of therapeutic proteins, such as monoclonal antibodies in the body of the patient, thus minimizing the tremendous costs associated with formulation, production, quality control and repeated administration of protein drugs (Deal and Balazs, 2015). For this purpose, intramuscular injections can be employed. Here, the physical availability of the target site allows efficient transfection by electroporation, but this is not possible for tumors or internal organs, such as the liver (Hollevoet and Declerck, 2017). For gene delivery into dividing cells, the DNA has the chance of entering the nucleus when the nuclear envelope is fragmented for mitosis. For post-mitotic cells, this is not an option. Furthermore, the passive diffusion of intact nanoparticles within the cytosol is minimal, as revealed by single-particle tracking (Remaut et al., 2014).

When the mechanism of DNA delivery by polyamine-containing agents was investigated, contributions of nuclear envelope permeabilization (consistent with their proposed action of endosomal permeabilization) and microtubule-directed transport were found, as well as dependency on cytosolic factors (Grandinetti and Reineke, 2012).

Apart from gene therapy approaches, other medications might also benefit from nuclear localization, such as anthracyclines, which exert their effects in the nucleus. The nuclear envelope is interspersed with nuclear pore complexes. They allow passive transport by diffusion of small molecules, but macromolecules require an active mechanism to be translocated because strings of phenylalanine- and glycine-rich repeats block the pores (Strambio-De-Castillia et al., 2010). For endogenous proteins, this is achieved by a nuclear localization sequence (NLS) which engages the importing machinery. In the realm of nanoparticles, gold nanoparticles of ~25 or 30 nm were modified with an NLS peptide to target them to the nucleus. Utilizing confocal microscopy, the particles were either found within the nucleus (Kang et al., 2010), or in the perinuclear region (Ali et al., 2017). Even such a perinuclear localization, however, would probably be quite efficient because it minimizes the necessary diffusion length of the cargo. With chitosan nanoparticles, heterogeneities in nuclear targeting deliveries were revealed: small particles of 25 nm entered the nucleus without the aid of an NLS. In non-malignant cell lines, 150 nm particles modified with low densities of NLS were more efficient at localizing nuclearly, whereas in a glioma cell line, this was most efficient for unmodified NPs, due to dysregulation of the nuclear import pathway (Tammam et al., 2017).

For nanoparticles, other approaches were also investigated, relying for instance on the HIV-derived Tat peptide which mediates nuclear import differently than the typical NLS-dependent mechanism and can deliver nanoparticles up to 90 nm into the nucleus (de la Fuente and Berry, 2005; Nitin et al., 2009). Functionalization of silica nanoparticles with the Tat peptide enabled them to enter the nucleus, whereas particles without Tat did not cross the nuclear envelope (Pan et al., 2013). Alternatively, modification of gold nanostars with the nucleolin-binding DNA aptamer AS1411 led to nuclear entry and morphological alterations of the nuclear envelope (Dam et al., 2012).

Finally, attempts at subcellular targeting are not limited to the nuclear compartment: Instead, approaches were developed, for example for targeting mitochondria, the endoplasmic reticulum or lysosomes, to deliver cargo precisely to its site of action (Jhaveri and Torchilin, 2016; Ma et al., 2016).

## 5. Conclusions

A principal attribute when discussing nanomedicine, emerging again and again at all levels and stages, is “heterogeneity.” As such, precisely delivering a nanotherapeutic agent exactly to where it needs to be to exert its maximal efficacy is a monumentally challenging and complex task, requiring the collaborative expertise from many different disciplines, including medicine, biology, chemistry, physics, the materials sciences, and engineering. Numerous barriers must be overcome before a cargo is finally delivered. Considerable progress has been made, but we are not quite there yet. Although there are strategies in place for tackling barriers during individual stages of this process, an integrated approach will require new and ingenious solutions to advance the field of nanomedicine beyond its current state. Current formulations often profoundly improve the toxicity profile of a drug, but do not substantially increase overall survival of a patient population (Petersen et al., 2016). Doxil, for instance, dramatically reduced the cumulative cardiotoxicity compared to free doxorubicin, whereas treatment efficacy was comparable between both groups in the treatment of metastatic breast cancer (Safra et al., 2000; O'Brien et al., 2004). To have a chance of entering clinical trials and becoming reliable tools, nanomedicines must be capable of overcoming the astounding complexity of their sites of action and the plethora of challenges these impose. At the same time, they must be sufficiently simple in their formulation and design to allow large-scale production. Unifying these opposing requirements will be difficult, but allow strides toward the advancement of science and medicine.

## Author Contributions

OT wrote the manuscript and prepared the figures. WW supervised the work, critically discussed the literature, and edited the manuscript.

### Conflict of Interest

The authors declare that the research was conducted in the absence of any commercial or financial relationships that could be construed as a potential conflict of interest.

## Abbreviations

ABC: accelerated blood clearance
CDM: 2-propionic-3-methylmaleic anhydride
CT: computed tomography
EPR: enhanced permeability and retention effect
hGH: human growth hormone
HIF: hypoxia-inducible factor
HIFU: high-intensity focused ultrasound
HPMA: poly(N-[2-hydroxypropyl] methacrylamide)
ID: injected dose
KS: Kaposi's sarcoma
LTSL: lyso-thermosensitive liposomes
MMP: matrix-metalloproteinase
Nbz: *o*-nitrobenzyl
NIR: near-infrared
NP: nanoparticle
PAcM: poly(N-acryloyl morpholine)
PAE: poly-β-aminoester
PAMAM: polyamidoamine
PCL: polycaprolactone
PDMA: poly(N,N-dimethylacrylamide)
PDT: photodynamic therapy
PEG: polyethylene glycol
PET: positron emission tomography
P-gp: P-glycoprotein
PLD: PEGylated liposomal doxorubicin
PLGA: poly(lactic-co-glycolic acid)
PMOX: poly(2-methyl-2-oxazoline)
POPE: 1-palmitoyl-2-oleoyl-sn-glycero-3-phosphoethanolamine
PPE: palmar-plantar erythrodysesthesia
PSar: polysarcosine
PVP: poly(vinylpyrrolidone)
RBC: red blood cell
RFA: radio frequency ablation
ROS: reactive oxygen species
scFv: single-chain variable fragment
SIRP: signal-regulatory protein
SPION: superparamagnetic iron oxide nanoparticle
TAM: tumor-associated macrophage
UCNP: upconversion nanoparticle
VEGF: vascular endothelial growth factor.

## References

[B1] Abu Lila A. S. Kiwada H. Ishida T. (2013). The accelerated blood clearance (ABC) phenomenon: clinical challenge and approaches to manage. J. Control. Release 172, 38–47. 10.1016/j.jconrel.2013.07.026 23933235

[B2] Ali M. R. K. Wu Y. Ghosh D. Do B. H. Chen K. Dawson M. R.. (2017). Nuclear Membrane-targeted gold nanoparticles inhibit cancer cell migration and invasion. ACS Nano 11, 3716–3726. 10.1021/acsnano.6b08345 28333438 PMC5519406

[B3] Alizadeh A. A. Aranda V. Bardelli A. Blanpain C. Bock C. Borowski C.. (2015). Toward understanding and exploiting tumor heterogeneity. Nat. Med. 21, 846–853. 10.1038/nm.3915 26248267 PMC4785013

[B4] Al-Jamal K. T. Bai J. Wang J. T.-W. Protti A. Southern P. Bogart L.. (2016). Magnetic drug targeting: preclinical *in vivo* studies, mathematical modeling, and extrapolation to humans. Nano Lett. 16, 5652–5660. 10.1021/acs.nanolett.6b02261 27541372

[B5] Anselmo A. C. Mitragotri S. (2016). Nanoparticles in the clinic. Bioeng. Transl. Med. 1, 10–29. 10.1002/btm2.10003 29313004 PMC5689513

[B6] Anselmo A. C. Mitragotri S. (2019). Nanoparticles in the clinic: an update. Bioeng. Transl. Med. 4:e10143. 10.1002/btm2.10143 31572799 PMC6764803

[B7] Armstrong J. K. Hempel G. Koling S. Chan L. S. Fisher T. Meiselman H. J.. (2007). Antibody against poly(ethylene glycol) adversely affects PEG-asparaginase therapy in acute lymphoblastic leukemia patients. Cancer 110, 103–111. 10.1002/cncr.22739 17516438

[B8] Armulik A. Abramsson A. Betsholtz C. (2005). Endothelial/pericyte interactions. Circ. Res. 97, 512–523. 10.1161/01.RES.0000182903.16652.d7 16166562

[B9] Attwell D. Mishra A. Hall C. N. O'Farrell F. M. Dalkara T. (2016). What is a pericyte? J. Cereb. Blood Flow Metab. 36, 451–455. 10.1177/0271678X15610340 26661200 PMC4759679

[B10] Azzi S. Hebda J. K. Gavard J. (2013). Vascular permeability and drug delivery in cancers. Front. Oncol. 3:211. 10.3389/fonc.2013.00211 23967403 PMC3744053

[B11] Bachmann M. F. Zinkernagel R. M. (1996). The influence of virus structure on antibody responses and virus serotype formation. Immunol. Today 17, 553–558. 10.1016/s0167-5699(96)10066-9 8991286

[B12] Baluk P. Morikawa S. Haskell A. Mancuso M. McDonald D. M. (2003). Abnormalities of basement membrane on blood vessels and endothelial sprouts in tumors. Am. J. Pathol. 163, 1801–1815. 10.1016/S0002-9440(10)63540-7 14578181 PMC1892429

[B13] Bangham A. D. Standish M. M. Watkins J. C. (1965). Diffusion of univalent ions across the lamellae of swollen phospholipids. J. Mol. Biol. 13, 238–252. 5859039 10.1016/s0022-2836(65)80093-6

[B14] Barenholz Y. (2012). Doxil® — the first FDA-approved nano-drug: lessons learned. J. Control. Release 160, 117–134. 10.1016/j.jconrel.2012.03.020 22484195

[B15] Bendele A. Seely J. Richey C. Sennello G. Shopp G. (1998). Short communication: renal tubular vacuolation in animals treated with polyethylene-glycol-conjugated proteins. Toxicol. Sci. 42, 152–157. 9579027 10.1006/toxs.1997.2396

[B16] Blanco E. Shen H. Ferrari M. (2015). Principles of nanoparticle design for overcoming biological barriers to drug delivery. Nat. Biotechnol. 33, 941–951. 10.1038/nbt.3330 26348965 PMC4978509

[B17] Bogart L. K. Pourroy G. Murphy C. J. Puntes V. Pellegrino T. Rosenblum D.. (2014). Nanoparticles for imaging, sensing, and therapeutic intervention. ACS Nano 8, 3107–3122. 10.1021/nn500962q 24641589 PMC4123720

[B18] Bolze F. Morath V. Bast A. Rink N. Schlapschy M. Mocek S.. (2016). Long-acting PASylated leptin ameliorates obesity by promoting satiety and preventing hypometabolism in leptin-deficient Lep(ob/ob) mice. Endocrinology 157, 233–244. 10.1210/en.2015-1519 26492472

[B19] Breibeck J. Skerra A. (2018). The polypeptide biophysics of proline/alanine-rich sequences (PAS): recombinant biopolymers with PEG-like properties. Biopolymers 109:e23069. 10.1002/bip.23069 29076532 PMC5813227

[B20] Brown E. J. Frazier W. A. (2001). Integrin-associated protein (CD47) and its ligands. Trends Cell Biol. 11, 130–135. 10.1016/S0962-8924(00)01906-1 11306274

[B21] Bulbake U. Doppalapudi S. Kommineni N. Khan W. (2017). Liposomal formulations in clinical use: an updated review. Pharmaceutics 9:E12. 10.3390/pharmaceutics9020012 28346375 PMC5489929

[B22] Bus T. Traeger A. Schubert U. S. (2018). The great escape: how cationic polyplexes overcome the endosomal barrier. J. Mater. Chem. B. 6, 6904–6918. 10.1039/C8TB00967H 32254575

[B23] Cabral H. Makino J. Matsumoto Y. Mi P. Wu H. Nomoto T.. (2015). Systemic targeting of lymph node metastasis through the blood vascular system by using size-controlled nanocarriers. ACS Nano 9, 4957–4967. 10.1021/nn5070259 25880444

[B24] Cabral H. Matsumoto Y. Mizuno K. Chen Q. Murakami M. Kimura M.. (2011). Accumulation of sub-100 nm polymeric micelles in poorly permeable tumours depends on size. Nat. Nanotechnol. 6, 815–823. 10.1038/nnano.2011.166 22020122

[B25] Chanan-Khan A. Szebeni J. Savay S. Liebes L. Rafique N. M. Alving C. R. (2003). Complement activation following first exposure to pegylated liposomal doxorubicin (Doxil®): possible role in hypersensitivity reactions. Ann. Oncol. 14, 1430–1437. 10.1093/annonc/mdg374 12954584

[B26] Charrois G. J. R. Allen T. M. (2003). Multiple injections of pegylated liposomal doxorubicin: pharmacokinetics and therapeutic activity. J. Pharmacol. Exp. Ther. 306, 1058–1067. 10.1124/jpet.103.053413 12808004

[B27] Chauhan V. P. Stylianopoulos T. Boucher Y. Jain R. K. (2011). Delivery of molecular and nanoscale medicine to tumors: transport barriers and strategies. Annu. Rev. Chem. Biomol. Eng. 2, 281–298. 10.1146/annurev-chembioeng-061010-114300 22432620

[B28] Chauhan V. P. Stylianopoulos T. Martin J. D. Popović Z. Chen O. Kamoun W. S.. (2012). Normalization of tumour blood vessels improves the delivery of nanomedicines in a size-dependent manner. Nat. Nanotechnol. 7, 383–388. 10.1038/nnano.2012.45 22484912 PMC3370066

[B29] Chen F. Ma K. Madajewski B. Zhuang L. Zhang L. Rickert K.. (2018). Ultrasmall targeted nanoparticles with engineered antibody fragments for imaging detection of HER2-overexpressing breast cancer. Nat. Commun. 9, 1–11. 10.1038/s41467-018-06271-5 30297810 PMC6175906

[B30] Cho K. Wang X. Nie S. Chen Z. G. Shin D. M. (2008). Therapeutic nanoparticles for drug delivery in cancer. Clin. Cancer Res. 14, 1310–1316. 10.1158/1078-0432.CCR-07-1441 18316549

[B31] Choi C. H. J. Alabi C. A. Webster P. Davis M. E. (2010). Mechanism of active targeting in solid tumors with transferrin-containing gold nanoparticles. Proc. Natl. Acad. Sci. U.S.A. 107, 1235–1240. 10.1073/pnas.0914140107 20080552 PMC2824286

[B32] Clark A. J. Davis M. E. (2015). Increased brain uptake of targeted nanoparticles by adding an acid-cleavable linkage between transferrin and the nanoparticle core. Proc. Natl. Acad. Sci. U.S.A. 112, 12486–12491. 10.1073/pnas.1517048112 26392563 PMC4603510

[B33] Clemons T. D. Singh R. Sorolla A. Chaudhari N. Hubbard A. Iyer K. S. (2018). Distinction between active and passive targeting of nanoparticles dictate their overall therapeutic efficacy. Langmuir 34, 15343–15349. 10.1021/acs.langmuir.8b02946 30441895

[B34] Cooley T. Henry D. Tonda M. Sun S. O'Connell M. Rackoff W. (2007). A randomized, double-blind study of pegylated liposomal doxorubicin for the treatment of AIDS-related Kaposi's sarcoma. Oncologist 12, 114–123. 10.1634/theoncologist.12-1-114 17227906

[B35] Coussens L. M. Zitvogel L. Palucka A. K. (2013). Neutralizing tumor-promoting chronic inflammation: a magic bullet? Science 339, 286–291. 10.1126/science.1232227 23329041 PMC3591506

[B36] Cupic K. I. Rennick J. J. Johnston A. P. Such G. K. (2018). Controlling endosomal escape using nanoparticle composition: current progress and future perspectives. Nanomedicine 14, 215–223. 10.2217/nnm-2018-0326 30511881

[B37] Dai Q. Walkey C. Chan W. C. W. (2014). Polyethylene glycol backfilling mitigates the negative impact of the protein corona on nanoparticle cell targeting. Angew. Chemie Int. Ed. 53, 5093–5096. 10.1002/anie.201309464 24700480

[B38] Dai Q. Wilhelm S. Ding D. Syed A. M. Sindhwani S. Zhang Y.. (2018). Quantifying the ligand-coated nanoparticle delivery to cancer cells in solid tumors. ACS Nano 12, 8423–8435. 10.1021/acsnano.8b03900 30016073

[B39] Dam D. H. M. Lee J. H. Sisco P. N. Co D. T. Zhang M. Wasielewski M. R.. (2012). Direct observation of nanoparticle–cancer cell nucleus interactions. ACS Nano 6, 3318–3326. 10.1021/nn300296p 22424173 PMC3337354

[B40] Danhier F. (2016). To exploit the tumor microenvironment: since the EPR effect fails in the clinic, what is the future of nanomedicine? J. Control. Release 244, 108–121. 10.1016/j.jconrel.2016.11.015 27871992

[B41] Davis M. E. Chen Z. G. Shin D. M. (2008). Nanoparticle therapeutics: an emerging treatment modality for cancer. Nat. Rev. Drug Discov. 7, 771–782. 10.1038/nrd2614 18758474

[B42] de la Fuente J. M. Berry C. C. (2005). Tat peptide as an efficient molecule to translocate gold nanoparticles into the cell nucleus. Bioconj. Chem. 16, 1176–1180. 10.1021/bc050033+ 16173795

[B43] Deal C. E. Balazs A. B. (2015). Engineering humoral immunity as prophylaxis or therapy. Curr. Opin. Immunol. 35, 113–122. 10.1016/j.coi.2015.06.014 26183209 PMC4553141

[B44] Dobrovolskaia M. A. McNeil S. E. (2007). Immunological properties of engineered nanomaterials. Nat. Nanotechnol. 2, 469–478. 10.1038/nnano.2007.223 18654343

[B45] Doherty G. J. McMahon H. T. (2009). Mechanisms of endocytosis. Ann. Rev. Biochem. 78, 857–902. 10.1146/annurev.biochem.78.081307.110540 19317650

[B46] Dominska M. Dykxhoorn D. M. (2010). Breaking down the barriers: siRNA delivery and endosome escape. J. Cell. Sci. 123, 1183–1189. 10.1242/jcs.066399 20356929

[B47] Dvorak H. F. (2003). How tumors make bad blood vessels and stroma. Am. J. Pathol. 162, 1747–1757. 10.1016/S0002-9440(10)64309-X 12759232 PMC1868128

[B48] Eales K. L. Hollinshead K. E. R. Tennant D. A. (2016). Hypoxia and metabolic adaptation of cancer cells. Oncogenesis 5:e190. 10.1038/oncsis.2015.50 26807645 PMC4728679

[B49] Eckes J. Schmah O. Siebers J. W. Groh U. Zschiedrich S. Rautenberg B.. (2011). Kinetic targeting of pegylated liposomal doxorubicin: a new approach to reduce toxicity during chemotherapy (CARL-trial). BMC Cancer 11:337. 10.1186/1471-2407-11-337 21816044 PMC3175222

[B50] El-Sawy H. S. Al-Abd A. M. Ahmed T. A. El-Say K. M. Torchilin V. P. (2018). Stimuli-responsive nano-architecture drug-delivery systems to solid tumor micromilieu: past, present, and future perspectives. ACS Nano 12, 10636–10664. 10.1021/acsnano.8b06104 30335963

[B51] Falvo E. Tremante E. Arcovito A. Papi M. Elad N. Boffi A.. (2016). Improved doxorubicin encapsulation and pharmacokinetics of ferritin–fusion protein nanocarriers bearing proline, serine, and alanine elements. Biomacromolecules 17, 514–522. 10.1021/acs.biomac.5b01446 26686226

[B52] Fang J. Nakamura H. Maeda H. (2011). The EPR effect: unique features of tumor blood vessels for drug delivery, factors involved, and limitations and augmentation of the effect. Adv. Drug Deliv. Rev. 63, 136–151. 10.1016/j.addr.2010.04.009 20441782

[B53] Fang J. S. Gillies R. D. Gatenby R. A. (2008). Adaptation to hypoxia and acidosis in carcinogenesis and tumor progression. Semin. Cancer Biol. 18, 330–337. 10.1016/j.semcancer.2008.03.011 18455429 PMC2953714

[B54] Fang R. H. Hu C.-M. J. Luk B. T. Gao W. Copp J. A. Tai Y.. (2014). Cancer cell membrane-coated nanoparticles for anticancer vaccination and drug delivery. Nano Lett. 14, 2181–2188. 10.1021/nl500618u 24673373 PMC3985711

[B55] Fischer D. Li Y. Ahlemeyer B. Krieglstein J. Kissel T. (2003). *In vitro* cytotoxicity testing of polycations: influence of polymer structure on cell viability and hemolysis. Biomaterials 24, 1121–1131. 10.1016/S0142-9612(02)00445-3 12527253

[B56] Francia V. Yang K. Deville S. Reker-Smit C. Nelissen I. Salvati A. (2019). Corona composition can affect the mechanisms cells use to internalize nanoparticles. ACS Nano 13, 11107–21. 10.1021/acsnano.9b03824 31525954 PMC6812477

[B57] Garay R. P. El-Gewely R. Armstrong J. K. Garratty G. Richette P. (2012). Antibodies against polyethylene glycol in healthy subjects and in patients treated with PEG-conjugated agents. Expert Opin. Drug. Deliv. 9, 1319–1323. 10.1517/17425247.2012.720969 22931049

[B58] Gerlowski L. E. Jain R. K. (1986). Microvascular permeability of normal and neoplastic tissues. Microvasc. Res. 31, 288–305. 2423854 10.1016/0026-2862(86)90018-x

[B59] Gilleron J. Querbes W. Zeigerer A. Borodovsky A. Marsico G. Schubert U.. (2013). Image-based analysis of lipid nanoparticle–mediated siRNA delivery, intracellular trafficking and endosomal escape. Nat. Biotechnol. 31, 638–646. 10.1038/nbt.2612 23792630

[B60] Giulimondi F. Digiacomo L. Pozzi D. Palchetti S. Vulpis E. Capriotti A. L.. (2019). Interplay of protein corona and immune cells controls blood residency of liposomes. Nat. Comm. 10, 1–11. 10.1038/s41467-019-11642-7 31417080 PMC6695391

[B61] Grandinetti G. Reineke T. M. (2012). Exploring the mechanism of plasmid DNA nuclear internalization with polymer-based vehicles. Mol. Pharm. 9, 2256–2267. 10.1021/mp300142d 22715912 PMC3601561

[B62] Gref R. Lück M. Quellec P. Marchand M. Dellacherie E. Harnisch S.. (2000). ‘Stealth' corona-core nanoparticles surface modified by polyethylene glycol (PEG): influences of the corona (PEG chain length and surface density) and of the core composition on phagocytic uptake and plasma protein adsorption. Colloids Surf. B 18, 301–313. 10.1016/S0927-7765(99)00156-3 10915952

[B63] Griffith D. E. Eagle G. Thomson R. Aksamit T. R. Hasegawa N. Morimoto K.. (2018). Amikacin liposome inhalation suspension for treatment-refractory lung disease caused by *Mycobacterium avium* complex (CONVERT). A prospective, open-label, randomized study. Am. J. Respir. Crit. Care Med. 198, 1559–1569. 10.1164/rccm.201807-1318OC 30216086

[B64] Hagendoorn J. Tong R. Fukumura D. Lin Q. Lobo J. Padera T. P.. (2006). Onset of abnormal blood and lymphatic vessel function and interstitial hypertension in early stages of carcinogenesis. Cancer Res. 66, 3360–3364. 10.1158/0008-5472.CAN-05-2655 16585153

[B65] Hanahan D. Weinberg R. A. (2011). Hallmarks of cancer: the next generation. Cell 144, 646–674. 10.1016/j.cell.2011.02.013 21376230

[B66] Hansen A. E. Petersen A. L. Henriksen J. R. Boerresen B. Rasmussen P. Elema D. R.. (2015). Positron emission tomography based elucidation of the enhanced permeability and retention effect in dogs with cancer using copper-64 liposomes. ACS Nano 9, 6985–6995. 10.1021/acsnano.5b01324 26022907

[B67] Hara E. Makino A. Kurihara K. Yamamoto F. Ozeki E. Kimura S. (2012). Pharmacokinetic change of nanoparticulate formulation “lactosome” on multiple administrations. Int. Immunopharmacol. 14, 261–266. 10.1016/j.intimp.2012.07.011 22841811

[B68] Haran G. Cohen R. Bar L. K. Barenholz Y. (1993). Transmembrane ammonium sulfate gradients in liposomes produce efficient and stable entrapment of amphipathic weak bases. Biochim. Biophys. Acta 1151, 201–215. 10.1016/0005-2736(93)90105-9 8373796

[B69] Hashizume H. Baluk P. Morikawa S. McLean J. W. Thurston G. Roberge S.. (2000). Openings between defective endothelial cells explain tumor vessel leakiness. Am. J. Pathol. 156, 1363–1380. 10.1016/S0002-9440(10)65006-7 10751361 PMC1876882

[B70] Hatakeyama H. Akita H. Harashima H. (2011). A multifunctional envelope type nano device (MEND) for gene delivery to tumours based on the EPR effect: a strategy for overcoming the PEG dilemma. Adv. Drug Deliv. Rev. 63, 152–160. 10.1016/j.addr.2010.09.001 20840859

[B71] Herrmann A. Nagao T. Zhang C. Lahtz C. Li Y.-J. Yue C.. (2019). An effective cell-penetrating antibody delivery platform. JCI Insight 4:e127474. 10.1172/jci.insight.127474 31341104 PMC6675557

[B72] Hobbs S. K. Monsky W. L. Yuan F. Roberts W. G. Griffith L. Torchilin V. P.. (1998). Regulation of transport pathways in tumor vessels: role of tumor type and microenvironment. Proc. Natl. Acad. Sci. U.S.A. 95, 4607–4612. 9539785 10.1073/pnas.95.8.4607 PMC22537

[B73] Hollevoet K. Declerck P. J. (2017). State of play and clinical prospects of antibody gene transfer. J. Transl. Med. 15:131. 10.1186/s12967-017-1234-4 28592330 PMC5463339

[B74] Hu C.-M. J. Fang R. H. Luk B. T. Chen K. N. H. Carpenter C. Gao W.. (2013). ‘Marker-of-self' functionalization of nanoscale particles through a top-down cellular membrane coating approach. Nanoscale 5, 2664–2668. 10.1039/c3nr00015j 23462967 PMC3667603

[B75] Hu C.-M. J. Zhang L. Aryal S. Cheung C. Fang R. H. Zhang L. (2011). Erythrocyte membrane-camouflaged polymeric nanoparticles as a biomimetic delivery platform. Proc. Natl. Acad. Sci. U.S.A. 108, 10980–10985. 10.1073/pnas.1106634108 21690347 PMC3131364

[B76] Hu Y. Hou Y. Wang H. Lu H. (2018). Polysarcosine as an alternative to PEG for therapeutic protein conjugation. Bioconj. Chem. 29, 2232–2238. 10.1021/acs.bioconjchem.8b00237 29863329

[B77] Hughes G. A. (2005). Nanostructure-mediated drug delivery. Nanomed. Nanotechnol. Biol. Med. 1, 22–30. 10.1016/j.nano.2004.11.009 17292054

[B78] Huotari J. Helenius A. (2011). Endosome maturation. EMBO J. 30, 3481–3500. 10.1038/emboj.2011.286 21878991 PMC3181477

[B79] Ishida T. Ichihara M. Wang X. Kiwada H. (2006). Spleen plays an important role in the induction of accelerated blood clearance of PEGylated liposomes. J. Control. Release 115, 243–250. 10.1016/j.jconrel.2006.08.001 17011060

[B80] Ishida T. Kiwada H. (2008). Accelerated blood clearance (ABC) phenomenon upon repeated injection of PEGylated liposomes. Int. J. Pharm. 354, 56–62. 10.1016/j.ijpharm.2007.11.005 18083313

[B81] Janssen Products (2019). Doxil Prescribing Information. Horsham, PA. Available online at: https://www.accessdata.fda.gov/drugsatfda_docs/label/2019/050718s055lbl.pdf

[B82] Jevševar S. Kunstelj M. Porekar V. G. (2010). PEGylation of therapeutic proteins. Biotech. J. 5, 113–128. 10.1002/biot.200900218 20069580

[B83] Jhaveri A. Torchilin V. (2016). Intracellular delivery of nanocarriers and targeting to subcellular organelles. Expert Opin. Drug. Deliv. 13, 49–70. 10.1517/17425247.2015.1086745 26358656

[B84] Jiang W. Huang Y. An Y. Kim B. Y. S. (2015). Remodeling tumor vasculature to enhance delivery of intermediate-sized nanoparticles. ACS Nano 9, 8689–8696. 10.1021/acsnano.5b02028 26212564

[B85] Jin Q. Deng Y. Chen X. Ji J. (2019). Rational design of cancer nanomedicine for simultaneous stealth surface and enhanced cellular uptake. ACS Nano 13, 954–977. 10.1021/acsnano.8b07746 30681834

[B86] Jokerst J. V. Lobovkina T. Zare R. N. Gambhir S. S. (2011). Nanoparticle PEGylation for imaging and therapy. Nanomedicine 6, 715–728. 10.2217/nnm.11.19 21718180 PMC3217316

[B87] Kalluri R. (2003). Basement membranes: structure, assembly and role in tumour angiogenesis. Nat. Rev. Cancer 3, 422–433. 10.1038/nrc1094 12778132

[B88] Kang B. Mackey M. A. El-Sayed M. A. (2010). Nuclear targeting of gold nanoparticles in cancer cells induces DNA damage, causing cytokinesis arrest and apoptosis. J. Am. Chem. Soc. 132, 1517–1519. 10.1021/ja9102698 20085324

[B89] Kano M. R. Komuta Y. Iwata C. Oka M. Shirai Y.-T. Morishita Y.. (2009). Comparison of the effects of the kinase inhibitors imatinib, sorafenib, and transforming growth factor-beta receptor inhibitor on extravasation of nanoparticles from neovasculature. Cancer Sci. 100, 173–180. 10.1111/j.1349-7006.2008.01003.x 19037999 PMC11158202

[B90] Kessenbrock K. Plaks V. Werb Z. (2010). Matrix metalloproteinases: regulators of the tumor microenvironment. Cell 141, 52–67. 10.1016/j.cell.2010.03.015 20371345 PMC2862057

[B91] Kierstead P. H. Okochi H. Venditto V. J. Chuong T. C. Kivimae S. Fréchet J. M. J.. (2015). The effect of polymer backbone chemistry on the induction of the accelerated blood clearance in polymer modified liposomes. J. Control. Release 213, 1–9. 10.1016/j.jconrel.2015.06.023 26093095 PMC4684485

[B92] Kim C. J. Hara E. Watabe N. Hara I. Kimura S. (2017). Modulation of immunogenicity of poly(sarcosine) displayed on various nanoparticle surfaces due to different physical properties. J. Peptide Sci. 23, 889–898. 10.1002/psc.3053 29110375

[B93] Kimura T. Takabatake Y. Takahashi A. Isaka Y. (2013). Chloroquine in cancer therapy: a double-edged sword of autophagy. Cancer Res. 73, 3–7. 10.1158/0008-5472.CAN-12-2464 23288916

[B94] Kozma G. T. Mészáros T. Vashegyi I. Fülöp T. Örfi E. Dézsi L.. (2019). Pseudo-anaphylaxis to polyethylene glycol (PEG)-coated liposomes: roles of anti-PEG IgM and complement activation in a porcine model of human infusion reactions. ACS Nano 13, 9315–9324. 10.1021/acsnano.9b03942 31348638

[B95] Krishnamurthy S. Muthukumaran P. Jayakumar M. K. G. Lisse D. Masurkar N. D. Xu C.. (2019). Surface protein engineering increases the circulation time of a cell membrane-based nanotherapeutic. Nanomed. Nanotechnol. Biol. Med. 18, 169–178. 10.1016/j.nano.2019.02.024 30853651

[B96] Kulkarni P. S. Haldar M. K. Nahire R. R. Katti P. Ambre A. H. Muhonen W. W.. (2014). MMP-9 responsive PEG cleavable nanovesicles for efficient delivery of chemotherapeutics to pancreatic cancer. Mol. Pharm. 11, 2390–2399. 10.1021/mp500108p 24827725 PMC4096225

[B97] Kunjachan S. Pola R. Gremse F. Theek B. Ehling J. Moeckel D.. (2014). Passive versus active tumor targeting using RGD- and NGR-modified polymeric nanomedicines. Nano Lett. 14, 972–981. 10.1021/nl404391r 24422585 PMC3940962

[B98] La-Beck N. M. Zamboni B. A. Gabizon A. Schmeeda H. Amantea M. Gehrig P. A.. (2012). Factors affecting the pharmacokinetics of pegylated liposomal doxorubicin in patients. Cancer Chemother. Pharmacol. 69, 43–50. 10.1007/s00280-011-1664-2 21590446

[B99] Lammers T. Kiessling F. Hennink W. E. Storm G. (2012a). Drug targeting to tumors: principles, pitfalls and (pre-) clinical progress. J. Control. Release 161, 175–187. 10.1016/j.jconrel.2011.09.063 21945285

[B100] Lammers T. Rizzo L. Y. Storm G. Kiessling F. (2012b). Personalized nanomedicine. Clin. Cancer Res. 18, 4889–4894. 10.1158/1078-0432.CCR-12-1414 22829203

[B101] Lancet J. E. Uy G. L. Cortes J. E. Newell L. F. Lin T. L. Ritchie E. K.. (2018). CPX-351 (cytarabine and daunorubicin) liposome for injection versus conventional cytarabine plus daunorubicin in older patients with newly diagnosed secondary acute myeloid leukemia. J. Clin. Oncol. 36, 2684–2692. 10.1200/JCO.2017.77.6112 30024784 PMC6127025

[B102] Lane L. A. Qian X. Smith A. M. Nie S. (2015). Physical chemistry of nanomedicine: understanding the complex behaviors of nanoparticles *in vivo*. Annu. Rev. Phys. Chem. 66, 521–547. 10.1146/annurev-physchem-040513-103718 25622189 PMC8590374

[B103] Lau K. H. A. Ren C. Sileika T. S. Park S. H. Szleifer I. Messersmith P. B. (2012). Surface-grafted polysarcosine as a peptoid antifouling polymer brush. Langmuir 28, 16099–16107. 10.1021/la302131n 23101930 PMC3530414

[B104] Laverman P. Carstens M. G. Boerman O. C. Dams E. T. M. Oyen W. J. G. van Rooijen N.. (2001). Factors affecting the accelerated blood clearance of polyethylene glycol-liposomes upon repeated injection. J. Pharmacol. Exp. Ther. 298, 607–612. Available online at: http://jpet.aspetjournals.org/content/298/2/607.long 11454922

[B105] Lee E. S. Gao Z. Bae Y. H. (2008). Recent progress in tumor pH targeting nanotechnology. J. Control. Release 132, 164–170. 10.1016/j.jconrel.2008.05.003 18571265 PMC2695946

[B106] Lee H. Gaddy D. Ventura M. Bernards N. de Souza R. Kirpotin D.. (2018). Companion diagnostic 64Cu-liposome positron emission tomography enables characterization of drug delivery to tumors and predicts response to cancer nanomedicines. Theranostics 8, 2300–2312. 10.7150/thno.21670 29721081 PMC5928891

[B107] Lee H. Shields A. F. Siegel B. A. Miller K. D. Krop I. Ma C. X.. (2017). 64Cu-MM-302 Positron emission tomography quantifies variability of enhanced permeability and retention of nanoparticles in relation to treatment response in patients with metastatic breast cancer. Clin. Cancer Res. 23, 4190–4202. 10.1158/1078-0432.CCR-16-3193 28298546 PMC6790129

[B108] Less J. R. Skalak T. C. Sevick E. M. Jain R. K. (1991). Microvascular architecture in a mammary carcinoma: branching patterns and vessel dimensions. Cancer Res. 51, 265–273. Available online at: http://cancerres.aacrjournals.org/content/51/1/265 1988088

[B109] Leu A. J. Berk D. A. Lymboussaki A. Alitalo K. Jain R. K. (2000). Absence of functional lymphatics within a murine sarcoma: a molecular and functional evaluation. Cancer Res. 60, 4324–4327. Available online at: https://cancerres.aacrjournals.org/content/60/16/4324.short 10969769

[B110] Levine B. Mizushima N. Virgin H. W. (2011). Autophagy in immunity and inflammation. Nature 469, 323–335. 10.1038/nature09782 21248839 PMC3131688

[B111] Li H. Liu Q. Crielaard B. J. de Vries J. W. Loznik M. Meng Z.. (2019). Fast, efficient, and targeted liposome delivery mediated by DNA hybridization. Adv. Healthc. Mat. 8:e1900389. 10.1002/adhm.201900389 31081288

[B112] Li H.-J. Du J.-Z. Du X.-J. Xu C.-F. Sun C.-Y. Wang H.-X.. (2016). Stimuli-responsive clustered nanoparticles for improved tumor penetration and therapeutic efficacy. Proc. Natl. Acad. Sci. U.S.A. 113, 4164–4169. 10.1073/pnas.1522080113 27035960 PMC4839420

[B113] Lu J. Li Z. Zink J. I. Tamanoi F. (2012). *In vivo* tumor suppression efficacy of mesoporous silica nanoparticles-based drug-delivery system: enhanced efficacy by folate modification. Nanomed. Nanotechnol. Biol. Med. 8, 212–220. 10.1016/j.nano.2011.06.002 21703996 PMC3221805

[B114] Lucky S. S. Soo K. C. Zhang Y. (2015). Nanoparticles in photodynamic therapy. Chem. Rev. 115, 1990–2042. 10.1021/cr5004198 25602130

[B115] Luo N. Weber J. K. Wang S. Luan B. Yue H. Xi X.. (2017). PEGylated graphene oxide elicits strong immunological responses despite surface passivation. Nat. Commun. 8:14537. 10.1038/ncomms14537 28233871 PMC5333105

[B116] Lyon P. C. Gray M. D. Mannaris C. Folkes L. K. Stratford M. Campo L.. (2018). Safety and feasibility of ultrasound-triggered targeted drug delivery of doxorubicin from thermosensitive liposomes in liver tumours (TARDOX): a single-centre, open-label, phase 1 trial. Lancet Oncol. 19, 1027–1039. 10.1016/S1470-2045(18)30332-2 30001990 PMC6073884

[B117] Ma X. Gong N. Zhong L. Sun J. Liang X.-J. (2016). Future of nanotherapeutics: targeting the cellular sub-organelles. Biomaterials 97, 10–21. 10.1016/j.biomaterials.2016.04.026 27155363

[B118] Maeda H. (2015). Toward a full understanding of the EPR effect in primary and metastatic tumors as well as issues related to its heterogeneity. Adv. Drug Deliv. Rev. 91, 3–6. 10.1016/j.addr.2015.01.002 25579058

[B119] Maeda H. Wu J. Sawa T. Matsumura Y. Hori K. (2000). Tumor vascular permeability and the EPR effect in macromolecular therapeutics: a review. J. Control. Release 65, 271–284. 10.1016/S0168-3659(99)00248-5 10699287

[B120] Magzoub M. Jin S. Verkman A. S. (2007). Enhanced macromolecule diffusion deep in tumors after enzymatic digestion of extracellular matrix collagen and its associated proteoglycan decorin. FASEB J. 22, 276–284. 10.1096/fj.07-9150com 17761521

[B121] Makino A. Kizaka-Kondoh S. Yamahara R. Hara I. Kanzaki T. Ozeki E.. (2009). Near-infrared fluorescence tumor imaging using nanocarrier composed of poly(l-lactic acid)-block-poly(sarcosine) amphiphilic polydepsipeptide. Biomaterials 30, 5156–5160. 10.1016/j.biomaterials.2009.05.046 19525007

[B122] Marusyk A. Polyak K. (2010). Tumor heterogeneity: causes and consequences. Biochim. Biophys. Acta 1805, 105–117. 10.1016/j.bbcan.2009.11.002 19931353 PMC2814927

[B123] Matea C. T. Mocan T. Tabaran F. Pop T. Mosteanu O. Puia C.. (2017). Quantum dots in imaging, drug delivery and sensor applications. Int. J. Nanomed. 12, 5421–5431. 10.2147/IJN.S138624 28814860 PMC5546783

[B124] Matsumoto Y. Nichols J. W. Toh K. Nomoto T. Cabral H. Miura Y.. (2016). Vascular bursts enhance permeability of tumour blood vessels and improve nanoparticle delivery. Nat. Nanotechnol. 11, 533–538. 10.1038/nnano.2015.342 26878143

[B125] Matsumura Y. Maeda H. (1986). A new concept for macromolecular therapeutics in cancer chemotherapy: Mechanism of tumoritropic accumulation of proteins and the antitumor agent smancs. Cancer Res. 46, 6387–6392. Available online at: https://cancerres.aacrjournals.org/content/46/12_Part_1/6387.short 2946403

[B126] McNeil S. E. (2016). Evaluation of nanomedicines: stick to the basics. Nat. Rev. Mater. 1:16073 10.1038/natrevmats.2016.73

[B127] Mebius R. E. Kraal G. (2005). Structure and function of the spleen. Nat. Rev. Immunol. 5:606. 10.1038/nri1669 16056254

[B128] Mendler C. T. Friedrich L. Laitinen I. Schlapschy M. Schwaiger M. Wester H.-J.. (2015). High contrast tumor imaging with radio-labeled antibody Fab fragments tailored for optimized pharmacokinetics via PASylation. mAbs 7, 96–109. 10.4161/19420862.2014.985522 25484039 PMC4622060

[B129] Mikada M. Sukhbaatar A. Miura Y. Horie S. Sakamoto M. Mori S.. (2017). Evaluation of the enhanced permeability and retention effect in the early stages of lymph node metastasis. Cancer Sci. 108, 846–852. 10.1111/cas.13206 28211204 PMC5448659

[B130] Miller K. Cortes J. Hurvitz S. A. Krop I. E. Tripathy D. Verma S. Riahi K.. (2016). HERMIONE: a randomized Phase 2 trial of MM-302 plus trastuzumab versus chemotherapy of physician's choice plus trastuzumab in patients with previously treated, anthracycline-naïve, HER2-positive, locally advanced/metastatic breast cancer. BMC Cancer 16:352. 10.1186/s12885-016-2385-z 27259714 PMC4893300

[B131] Miller S. M. Simon R. J. Ng S. Zuckermann R. N. Kerr J. M. Moos W. H. (1995). Comparison of the proteolytic susceptibilities of homologous L-amino acid, D-amino acid, and N-substituted glycine peptide and peptoid oligomers. Drug Dev. Res. 35, 20–32.

[B132] Mima Y. Abu Lila A. S. Shimizu T. Ukawa M. Ando H. Kurata Y.. (2017). Ganglioside inserted into PEGylated liposome attenuates anti-PEG immunity. J. Control. Release 250, 20–26. 10.1016/j.jconrel.2017.01.040 28179196

[B133] Mishra S. Webster P. Davis M. E. (2004). PEGylation significantly affects cellular uptake and intracellular trafficking of non-viral gene delivery particles. Eur. J. Cell Biol. 83, 97–111. 10.1078/0171-9335-00363 15202568

[B134] Morath V. Bolze F. Schlapschy M. Schneider S. Sedlmayer F. Seyfarth K.. (2015). PASylation of murine leptin leads to extended plasma half-life and enhanced *in vivo* Efficacy. Mol. Pharm. 12, 1431–1442. 10.1021/mp5007147 25811325

[B135] Morikawa S. Baluk P. Kaidoh T. Haskell A. Jain R. K. McDonald D. M. (2002). Abnormalities in pericytes on blood vessels and endothelial sprouts in tumors. Am. J. Pathol. 160, 985–1000. 10.1016/S0002-9440(10)64920-6 11891196 PMC1867175

[B136] Mura S. Nicolas J. Couvreur P. (2013). Stimuli-responsive nanocarriers for drug delivery. Nat. Mater. 12, 991–1003. 10.1038/nmat3776 24150417

[B137] Nagy J. A. Chang S.-H. Shih S.-C. Dvorak A. M. Dvorak H. F. (2010). Heterogeneity of the tumor vasculature. Sem. Throm. Hemost. 36, 321–331. 10.1055/s-0030-1253454 20490982 PMC3278036

[B138] Needham D. Anyarambhatla G. Kong G. Dewhirst M. W. (2000). A new temperature-sensitive liposome for use with mild hyperthermia: characterization and testing in a human tumor xenograft model. Cancer Res. 60, 1197–1201. Available online at: https://cancerres.aacrjournals.org/content/60/5/1197 10728674

[B139] Ngoune R. Peters A. von Elverfeldt D. Winkler K. Pütz G. (2016). Accumulating nanoparticles by EPR: a route of no return. J. Control. Release 238, 58–70. 10.1016/j.jconrel.2016.07.028 27448444

[B140] Nitin N. LaConte L. Rhee W. J. Bao G. (2009). Tat peptide is capable of importing large nanoparticles across nuclear membrane in digitonin permeabilized cells. Ann. Biomed. Eng. 37, 2018–2027. 10.1007/s10439-009-9768-0 19657743 PMC2745486

[B141] Noguchi Y. Wu J. Duncan R. Strohalm J. Ulbrich K. Akaike T.. (1998). Early phase tumor accumulation of macromolecules: a great difference in clearance rate between tumor and normal tissues. Jpn. J. Cancer Res. 89, 307–314. 9600125 10.1111/j.1349-7006.1998.tb00563.x PMC5921799

[B142] Northfelt D. W. Dezube B. J. Thommes J. A. Miller B. J. Fischl M. A. Friedman-Kien A.. (1998). Pegylated-liposomal doxorubicin versus doxorubicin, bleomycin, and vincristine in the treatment of AIDS-related Kaposi's sarcoma: results of a randomized phase III clinical trial. J. Clin. Oncol. 16, 2445–2451. 9667262 10.1200/JCO.1998.16.7.2445

[B143] Northfelt D. W. Martin F. J. Working P. Volberding P. A. Russell J. Newman M.. (1996). Doxorubicin encapsulated in liposomes containing surface-bound polyethylene glycol: pharmacokinetics, tumor localization, and safety in patients with AIDS-related Kaposi's sarcoma. J. Clin. Oncol. 36, 55–63. 8932544 10.1002/j.1552-4604.1996.tb04152.x

[B144] O'Brien M. E. R. Wigler N. Inbar M. Rosso R. Grischke E. Santoro A. (2004). Reduced cardiotoxicity and comparable efficacy in a phase III trial of pegylated liposomal doxorubicin HCl (CAELYX™/Doxil®) versus conventional doxorubicin for first-line treatment of metastatic breast cancer. Ann. Oncol. 15, 440–449. 10.1093/annonc/mdh097 14998846

[B145] Ogun S. A. Dumon-Seignovert L. Marchand J.-B. Holder A. A. Hill F. (2008). The oligomerization domain of C4-binding protein (C4bp) acts as an adjuvant, and the fusion protein comprised of the 19-kilodalton merozoite surface protein 1 fused with the murine C4bp domain protects mice against malaria. Infect. Immun. 76, 3817–3823. 10.1128/IAI.01369-07 18474650 PMC2493234

[B146] Olive K. P. Jacobetz M. A. Davidson C. J. Gopinathan A. McIntyre D. Honess D.. (2009). Inhibition of Hedgehog signaling enhances delivery of chemotherapy in a mouse model of pancreatic cancer. Science 324, 1457–1461. 10.1126/science.1171362 19460966 PMC2998180

[B147] Padera T. P. Kadambi A. di Tomaso E. Carreira C. M. Brown E. B. Boucher Y.. (2002). Lymphatic metastasis in the absence of functional intratumor lymphatics. Science 296, 1883–1886. 10.1126/science.1071420 11976409

[B148] Padera T. P. Stoll B. R. Tooredman J. B. Capen D. di Tomaso E. Jain R. K. (2004). Pathology: cancer cells compress intratumour vessels. Nature 427:695. 10.1038/427695a 14973470

[B149] Pan L. Liu J. He Q. Wang L. Shi J. (2013). Overcoming multidrug resistance of cancer cells by direct intranuclear drug delivery using TAT-conjugated mesoporous silica nanoparticles. Biomaterials 34, 2719–2730. 10.1016/j.biomaterials.2012.12.040 23337327

[B150] Parhi P. Mohanty C. Sahoo S. K. (2012). Nanotechnology-based combinational drug delivery: an emerging approach for cancer therapy. Drug Discov. Today 17, 1044–1052. 10.1016/j.drudis.2012.05.010 22652342

[B151] Pelaz B. del Pino P. Maffre P. Hartmann R. Gallego M. Rivera-Fernández S.. (2015). Surface functionalization of nanoparticles with polyethylene glycol: effects on protein adsorption and cellular uptake. ACS Nano 9, 6996–7008. 10.1021/acsnano.5b01326 26079146

[B152] Peng F. Setyawati M. I. Tee J. K. Ding X. Wang J. Nga M. E.. (2019). Nanoparticles promote *in vivo* breast cancer cell intravasation and extravasation by inducing endothelial leakiness. Nat. Nanotechnol. 14:279. 10.1038/s41565-018-0356-z 30692675

[B153] Petersen G. H. Alzghari S. K. Chee W. Sankari S. S. La-Beck N. M. (2016). Meta-analysis of clinical and preclinical studies comparing the anticancer efficacy of liposomal versus conventional non-liposomal doxorubicin. J. Control. Release 232, 255–264. 10.1016/j.jconrel.2016.04.028 27108612

[B154] Pirollo K. F. Chang E. H. (2008). Does a targeting ligand influence nanoparticle tumor localization or uptake? Trends Biotechnol. 26, 552–558. 10.1016/j.tibtech.2008.06.007 18722682

[B155] Pluen A. Boucher Y. Ramanujan S. McKee T. D. Gohongi T. di Tomaso E.. (2001). Role of tumor–host interactions in interstitial diffusion of macromolecules: cranial vs. subcutaneous tumors. Proc. Natl. Acad. Sci. U.S.A. 98, 4628–4633. 10.1073/pnas.081626898 11274375 PMC31885

[B156] Prijic S. Sersa G. (2011). Magnetic nanoparticles as targeted delivery systems in oncology. Radiol. Oncol. 45, 1–16. 10.2478/v10019-011-0001-z 22933928 PMC3423716

[B157] Radu O. Pantanowitz L. (2013). Kaposi sarcoma. Arch. Pathol. Lab. Med. 137, 289–294. 10.5858/arpa.2012-0101-RS 23368874

[B158] Rees P. Wills J. W. Brown M. R. Barnes C. M. Summers H. D. (2019). The origin of heterogeneous nanoparticle uptake by cells. Nat. Commun. 10, 1–8. 10.1038/s41467-019-10112-4 31138801 PMC6538724

[B159] Remaut K. Lucas B. Braeckmans K. Demeester J. De Smedt S. C. (2007). Pegylation of liposomes favours the endosomal degradation of the delivered phosphodiester oligonucleotides. J. Control. Release 117, 256–266. 10.1016/j.jconrel.2006.10.029 17188777

[B160] Remaut K. Oorschot V. Braeckmans K. Klumperman J. De Smedt S. C. (2014). Lysosomal capturing of cytoplasmic injected nanoparticles by autophagy: an additional barrier to non viral gene delivery. J. Control. Release 195, 29–36. 10.1016/j.jconrel.2014.08.002 25125327

[B161] Roberts R. Al-Jamal W. T. Whelband M. Thomas P. Jefferson M. van den Bossche J.. (2013). Autophagy and formation of tubulovesicular autophagosomes provide a barrier against nonviral gene delivery. Autophagy 9, 667–682. 10.4161/auto.23877 23422759 PMC3669178

[B162] Rosenblum D. Joshi N. Tao W. Karp J. M. Peer D. (2018). Progress and challenges towards targeted delivery of cancer therapeutics. Nat. Commun. 9, 1–12. 10.1038/s41467-018-03705-y 29650952 PMC5897557

[B163] Rudge S. Peterson C. Vessely C. Koda J. Stevens S. Catterall L. (2001). Adsorption and desorption of chemotherapeutic drugs from a magnetically targeted carrier (MTC). J. Control. Release 74, 335–340. 10.1016/S0168-3659(01)00344-3 11489515

[B164] Rudmann D. G. Alston J. T. Hanson J. C. Heidel S. (2013). High molecular weight polyethylene glycol cellular distribution and PEG-associated cytoplasmic vacuolation is molecular weight dependent and does not require conjugation to proteins. Toxicol. Pathol. 41, 970–983. 10.1177/0192623312474726 23788571

[B165] Sabnani M. K. Rajan R. Rowland B. Mavinkurve V. Wood L. M. Gabizon A. A.. (2015). Liposome promotion of tumor growth is associated with angiogenesis and inhibition of antitumor immune responses. Nanomed. Nanotechnol. Biol. Med. 11, 259–262. 10.1016/j.nano.2014.08.010 25200609

[B166] Safra T. Muggia F. Jeffers S. Tsao-Wei D. D. Groshen S. Lyass O.. (2000). Pegylated liposomal doxorubicin (doxil): reduced clinical cardiotoxicity in patients reaching or exceeding cumulative doses of 500 mg/m^2^. Ann. Oncol. 11, 1029–1033. 10.1023/A:1008365716693 11038041

[B167] Sahay G. Alakhova D. Y. Kabanov A. V. (2010). Endocytosis of nanomedicines. J. Control. Release 145, 182–195. 10.1016/j.jconrel.2010.01.036 20226220 PMC2902597

[B168] Sahay G. Querbes W. Alabi C. Eltoukhy A. Sarkar S. Zurenko C.. (2013). Efficiency of siRNA delivery by lipid nanoparticles is limited by endocytic recycling. Nat. Biotechnol. 31, 653–658. 10.1038/nbt.2614 23792629 PMC3814166

[B169] Salvati A. Pitek A. S. Monopoli M. P. Prapainop K. Bombelli F. B. Hristov D. R.. (2013). Transferrin-functionalized nanoparticles lose their targeting capabilities when a biomolecule corona adsorbs on the surface. Nat. Nanotechnol. 8, 137–143. 10.1038/nnano.2012.237 23334168

[B170] Saneja A. Dubey R. D. Alam N. Khare V. Gupta P. N. (2014). Co-formulation of P-glycoprotein substrate and inhibitor in nanocarriers: an emerging strategy for cancer chemotherapy. Curr. Cancer Drug Targets 14, 419–433. 10.2174/1568009614666140407112034 24720364

[B171] Sarisozen C. Abouzeid A. H. Torchilin V. P. (2014). The effect of co-delivery of paclitaxel and curcumin by transferrin-targeted PEG-PE-based mixed micelles on resistant ovarian cancer in 3-D spheroids and *in vivo* tumors. Eur. J. Pharm. Biopharm. 88, 539–550. 10.1016/j.ejpb.2014.07.001 25016976 PMC4252620

[B172] Schlapschy M. Binder U. Börger C. Theobald I. Wachinger K. Kisling S.. (2013). PASylation: a biological alternative to PEGylation for extending the plasma half-life of pharmaceutically active proteins. Protein Eng. Des. Sel. 26, 489–501. 10.1093/protein/gzt023 23754528 PMC3715784

[B173] Schöttler S. Becker G. Winzen S. Steinbach T. Mohr K. Landfester K.. (2016). Protein adsorption is required for stealth effect of poly(ethylene glycol)- and poly(phosphoester)-coated nanocarriers. Nat. Nanotechnol. 11, 372–377. 10.1038/nnano.2015.330 26878141

[B174] Setyawati M. I. Tay C. Y. Bay B. H. Leong D. T. (2017). Gold nanoparticles induced endothelial leakiness depends on particle size and endothelial cell origin. ACS Nano 11, 5020–5030. 10.1021/acsnano.7b01744 28422481

[B175] Shapiro B. Kulkarni S. Nacev A. Muro S. Stepanov P. Y. Weinberg I. N. (2015). Open challenges in magnetic drug targeting. WIREs Nanomed. Nanobiotechnol. 7, 446–457. 10.1002/wnan.1311 25377422 PMC4397114

[B176] Silverman L. Barenholz Y. (2015). *In vitro* experiments showing enhanced release of doxorubicin from Doxil® in the presence of ammonia may explain drug release at tumor site. Nanomed. Nanotechnol. Biol. Med. 11, 1841–1850. 10.1016/j.nano.2015.06.007 26115641

[B177] Smith N. R. Baker D. Farren M. Pommier A. Swann R. Wang X.. (2013). Tumor stromal architecture can define the intrinsic tumor response to VEGF-targeted therapy. Clin. Cancer Res. 19, 6943–6956. 10.1158/1078-0432.CCR-13-1637 24030704

[B178] Smith S. A. Selby L. I. Johnston A. P. R. Such G. K. (2019). The endosomal escape of nanoparticles: toward more efficient cellular delivery. Bioconj. Chem. 30, 263–272. 10.1021/acs.bioconjchem.8b00732 30452233

[B179] Sofuni A. Iijima H. Moriyasu F. Nakayama D. Shimizu M. Nakamura K.. (2005). Differential diagnosis of pancreatic tumors using ultrasound contrast imaging. J. Gastrroenterol. 40, 518–525. 10.1007/s00535-005-1578-z 15942718

[B180] Stacker S. A. Williams S. P. Karnezis T. Shayan R. Fox S. B. Achen M. G. (2014). Lymphangiogenesis and lymphatic vessel remodelling in cancer. Nat. Rev. Cancer 14, 159–172. 10.1038/nrc3677 24561443

[B181] Stan R. V. (2007). Endothelial stomatal and fenestral diaphragms in normal vessels and angiogenesis. J. Cell. Mol. Med. 11, 621–643. 10.1111/j.1582-4934.2007.00075.x 17760829 PMC3823246

[B182] Strambio-De-Castillia C. Niepel M. Rout M. P. (2010). The nuclear pore complex: bridging nuclear transport and gene regulation. Nat. Rev. Mol. Cell Biol. 11, 490–501. 10.1038/nrm2928 20571586

[B183] Suk J. S. Xu Q. Kim N. Hanes J. Ensign L. M. (2016). PEGylation as a strategy for improving nanoparticle-based drug and gene delivery. Adv. Drug Deliv. Rev. 99, 28–51. 10.1016/j.addr.2015.09.012 26456916 PMC4798869

[B184] Szebeni J. Baranyi L. Savay S. Milosevits J. Bunger R. Laverman P.. (2002). Role of complement activation in hypersensitivity reactions to doxil and hynic peg liposomes: experimental and clinical studies. J. Liposome Res. 12, 165–172. 10.1081/LPR-120004790 12604051

[B185] Szebeni J. Simberg D. González-Fernández Á. Barenholz Y. Dobrovolskaia M. A. (2018). Roadmap and strategy for overcoming infusion reactions to nanomedicines. Nat. Nanotechnol. 13, 1100–1108. 10.1038/s41565-018-0273-1 30348955 PMC6320688

[B186] Tak W. Y. Lin S.-M. Wang Y. Zheng J. Vecchione A. Park S. Y.. (2018). Phase III HEAT study adding Lyso-thermosensitive liposomal doxorubicin to radiofrequency ablation in patients with unresectable hepatocellular carcinoma lesions. Clin. Cancer Res. 24, 73–83. 10.1158/1078-0432.CCR-16-2433 29018051

[B187] Tammam S. N. Azzazy H. M. E. Lamprecht A. (2017). The effect of nanoparticle size and NLS density on nuclear targeting in cancer and normal cells; impaired nuclear import and aberrant nanoparticle intracellular trafficking in glioma. J. Control. Release 253, 30–36. 10.1016/j.jconrel.2017.02.029 28254629

[B188] Tang J. Howard B. C. Mahler M. S. Thurecht J. K. Huang L. Ping Xu Z. (2018). Enhanced delivery of siRNA to triple negative breast cancer cells *in vitro* and *in vivo* through functionalizing lipid-coated calcium phosphate nanoparticles with dual target ligands. Nanoscale 10, 4258–4266. 10.1039/C7NR08644J 29436549

[B189] Tang J. Zhang L. Gao H. Liu Y. Zhang Q. Ran R.. (2016). Co-delivery of doxorubicin and P-gp inhibitor by a reduction-sensitive liposome to overcome multidrug resistance, enhance anti-tumor efficiency and reduce toxicity. Drug Deliv. 23, 1130–1143. 10.3109/10717544.2014.990651 25491241

[B190] Tavares A. J. Poon W. Zhang Y.-N. Dai Q. Besla R. Ding D.. (2017). Effect of removing Kupffer cells on nanoparticle tumor delivery. Proc. Natl. Acad. Sci. U.S.A. 114, E10871–E10880. 10.1073/pnas.1713390114 29208719 PMC5754793

[B191] Tay C. Y. Setyawati M. I. Leong D. T. (2017). Nanoparticle density: a critical biophysical regulator of endothelial permeability. ACS Nano 11, 2764–2772. 10.1021/acsnano.6b07806 28287706

[B192] Tietze R. Lyer S. Dürr S. Struffert T. Engelhorn T. Schwarz M.. (2013). Efficient drug-delivery using magnetic nanoparticles — biodistribution and therapeutic effects in tumour bearing rabbits. Nanomed. Nanotechnol. Biol. Med. 9, 961–971. 10.1016/j.nano.2013.05.001 23669367

[B193] Tietze R. Zaloga J. Unterweger H. Lyer S. Friedrich R. P. Janko C.. (2015). Magnetic nanoparticle-based drug delivery for cancer therapy. Biochem. Biophys. Res. Commun. 468, 463–470. 10.1016/j.bbrc.2015.08.022 26271592

[B194] Tong R. T. Boucher Y. Kozin S. V. Winkler F. Hicklin D. J. Jain R. K. (2004). Vascular normalization by vascular endothelial growth factor receptor 2 blockade induces a pressure gradient across the vasculature and improves drug penetration in tumors. Cancer Res. 64, 3731–3736. 10.1158/0008-5472.CAN-04-0074 15172975

[B195] Tsukigawa K. Liao L. Nakamura H. Fang J. Greish K. Otagiri M.. (2015). Synthesis and therapeutic effect of styrene–maleic acid copolymer-conjugated pirarubicin. Cancer Sci. 106, 270–278. 10.1111/cas.12592 25529761 PMC4376435

[B196] Udhrain A. Skubitz K. M. Northfelt D. W. (2007). Pegylated liposomal doxorubicin in the treatment of AIDS-related Kaposi's sarcoma. Int. J. Nanomed. 2, 345–352. Available online at: https://www.dovepress.com/pegylated-liposomal-doxorubicin-in-the-treatment-of-aids-related-kapos-peer-reviewed-article-IJN# 18019833 PMC2676669

[B197] Ulbrich K. Hekmatara T. Herbert E. Kreuter J. (2009). Transferrin- and transferrin-receptor-antibody-modified nanoparticles enable drug delivery across the blood–brain barrier (BBB). Eur. J. Pharm. Biopharm. 71, 251–256. 10.1016/j.ejpb.2008.08.021 18805484

[B198] Vermeulen L. M. P. De Smedt S. C. Remaut K. Braeckmans K. (2018). The proton sponge hypothesis: fable or fact? Eur. J. Pharm. Biopharm. 129, 184–190. 10.1016/j.ejpb.2018.05.034 29859281

[B199] Vu V. P. Gifford G. B. Chen F. Benasutti H. Wang G. Groman E. V. (2019). Immunoglobulin deposition on biomolecule corona determines complement opsonization efficiency of preclinical and clinical nanoparticles. Nat. Nanotechnol. 14, 260–268. 10.1038/s41565-018-0344-3 30643271 PMC6402998

[B200] Wan Y. Han J. Fan G. Zhang Z. Gong T. Sun X. (2013). Enzyme-responsive liposomes modified adenoviral vectors for enhanced tumor cell transduction and reduced immunogenicity. Biomaterials 34, 3020–3030. 10.1016/j.biomaterials.2012.12.051 23360783

[B201] Wang Y. Dou L. He H. Zhang Y. Shen Q. (2014). Multifunctional nanoparticles as nanocarrier for vincristine sulfate delivery to overcome tumor multidrug resistance. Mol. Pharm. 11, 885–894. 10.1021/mp400547u 24483832

[B202] Wang Y. Kohane D. S. (2017). External triggering and triggered targeting strategies for drug delivery. Nat. Rev. Mater. 2:17020 10.1038/natrevmats.2017.20

[B203] Wang-Gillam A. Hubner R. A. Siveke J. T. Von Hoff D. D. Belanger B. de Jong F. A.. (2019). NAPOLI-1 phase 3 study of liposomal irinotecan in metastatic pancreatic cancer: final overall survival analysis and characteristics of long-term survivors. Eur. J. Cancer 108, 78–87. 10.1016/j.ejca.2018.12.007 30654298

[B204] Wang-Gillam A. Li C.-P. Bodoky G. Dean A. Shan Y.-S. Jameson G.. (2016). Nanoliposomal irinotecan with fluorouracil and folinic acid in metastatic pancreatic cancer after previous gemcitabine-based therapy (NAPOLI-1): a global, randomised, open-label, phase 3 trial. Lancet 387, 545–557. 10.1016/S0140-6736(15)00986-1 26615328

[B205] Weber B. Seidl C. Schwiertz D. Scherer M. Bleher S. Süss R.. (2016). Polysarcosine-based lipids: from lipopolypeptoid micelles to stealth-like lipids in langmuir blodgett monolayers. Polymers 8:427. 10.3390/polym8120427 30974703 PMC6432249

[B206] Webster R. Didier E. Harris P. Siegel N. Stadler J. Tilbury L.. (2007). PEGylated proteins: evaluation of their safety in the absence of definitive metabolism studies. Drug Metab. Dispos. 35, 9–16. 10.1124/dmd.106.012419 17020954

[B207] Webster R. Elliott V. Park B. K. Walker D. Hankin M. Taupin P. (2009). “PEG and PEG conjugates toxicity: towards an understanding of the toxicity of PEG and its relevance to PEGylated biologicals,” in PEGylated Protein Drugs: Basic Science and Clinical Applications, Milestones in Drug Therapy, ed Veronese F. M. (Basel: Birkhäuser Basel), 127–146.

[B208] Wei X. Shamrakov D. Nudelman S. Peretz-Damari S. Nativ-Roth E. Regev O.. (2018). Cardinal role of intraliposome doxorubicin-sulfate nanorod crystal in doxil properties and performance. ACS Omega 3, 2508–2517. 10.1021/acsomega.7b01235 30023837 PMC6044617

[B209] Wei Y. Gu X. Cheng L. Meng F. Storm G. Zhong Z. (2019). Low-toxicity transferrin-guided polymersomal doxorubicin for potent chemotherapy of orthotopic hepatocellular carcinoma *in vivo*. Acta Biomat. 92, 196–204. 10.1016/j.actbio.2019.05.034 31102765

[B210] Weiss A. C. G. Kelly H. G. Faria M. Besford Q. A. Wheatley A. K. Ang C.-S.. (2019). Link between low-fouling and stealth: a whole blood biomolecular corona and cellular association analysis on nanoengineered particles. ACS Nano 13, 4980–4991. 10.1021/acsnano.9b00552 30998312

[B211] Wen S. Zhou J. Zheng K. Bednarkiewicz A. Liu X. Jin D. (2018). Advances in highly doped upconversion nanoparticles. Nat. Commun. 9, 1–12. 10.1038/s41467-018-04813-5 29925838 PMC6010470

[B212] Wenande E. Garvey L. H. (2016). Immediate-type hypersensitivity to polyethylene glycols: a review. Clin. Exp. Allergy 46, 907–922. 10.1111/cea.12760 27196817

[B213] Wiley D. T. Webster P. Gale A. Davis M. E. (2013). Transcytosis and brain uptake of transferrin-containing nanoparticles by tuning avidity to transferrin receptor. Proc. Natl. Acad. Sci. U.S.A. 110, 8662–8667. 10.1073/pnas.1307152110 23650374 PMC3666717

[B214] Wilhelm S. Tavares A. J. Dai Q. Ohta S. Audet J. Dvorak H. F. (2016). Analysis of nanoparticle delivery to tumours. Nat. Rev. Mater. 1:16014 10.1038/natrevmats.2016.14

[B215] Wu J. Akaike T. Maeda H. (1998). Modulation of enhanced vascular permeability in tumors by a Bradykinin antagonist, a cyclooxygenase inhibitor, and a nitric oxide scavenger. Cancer Res. 58, 159–165. Available online at: https://cancerres.aacrjournals.org/content/58/1/159.long 9426072

[B216] Xia Y. Schlapschy M. Morath V. Roeder N. Vogt E. I. Stadler D.. (2019). PASylated interferon α efficiently suppresses hepatitis B virus and induces anti-HBs seroconversion in HBV-transgenic mice. Antiviral Res. 161, 134–143. 10.1016/j.antiviral.2018.11.003 30439382

[B217] Xu S. Zhu X. Zhang C. Huang W. Zhou Y. Yan D. (2018). Oxygen and Pt(II) self-generating conjugate for synergistic photo-chemo therapy of hypoxic tumor. Nat. Commun. 9, 1–9. 10.1038/s41467-018-04318-1 29795534 PMC5967320

[B218] Yankai Z. Rong Y. Yi H. Wentao L. Rongyue C. Ming Y.. (2006). Ten tandem repeats of beta-hCG 109-118 enhance immunogenicity and anti-tumor effects of beta-hCG C-terminal peptide carried by mycobacterial heat-shock protein HSP65. Biochem. Biophys. Res. Commun. 345, 1365–1371. 10.1016/j.bbrc.2006.05.022 16725110

[B219] Yin H. Kanasty R. L. Eltoukhy A. A. Vegas A. J. Dorkin J. R. Anderson D. G. (2014). Non-viral vectors for gene-based therapy. Nat. Rev. Gen. 15, 541–555. 10.1038/nrg3763 25022906

[B220] Yu M. Wu J. Shi J. Farokhzad O. C. (2016). Nanotechnology for protein delivery: overview and perspectives. J. Control. Release 240, 24–37. 10.1016/j.jconrel.2015.10.012 26458789 PMC4833694

[B221] Yuan F. Dellian M. Fukumura D. Leunig M. Berk D. A. Torchilin V. P.. (1995). Vascular permeability in a human tumor xenograft: molecular size dependence and cutoff size. Cancer Res. 55, 3752–3756. Available online at: https://cancerres.aacrjournals.org/content/55/17/3752.long 7641188

[B222] Zhang C.-G. Zhu W.-J. Liu Y. Yuan Z.-Q. Yang S.-D. Chen W.-L.. (2016). Novel polymer micelle mediated co-delivery of doxorubicin and P-glycoprotein siRNA for reversal of multidrug resistance and synergistic tumor therapy. Sci. Rep. 6:23859. 10.1038/srep23859 27030638 PMC4814909

[B223] Zhang S. Gao H. Bao G. (2015). Physical principles of nanoparticle cellular endocytosis. ACS Nano 9, 8655–8671. 10.1021/acsnano.5b03184 26256227 PMC5681865

[B224] Zhang X. Dong Y. Zeng X. Liang X. Li X. Tao W.. (2014). The effect of autophagy inhibitors on drug delivery using biodegradable polymer nanoparticles in cancer treatment. Biomaterials 35, 1932–1943. 10.1016/j.biomaterials.2013.10.034 24315578

[B225] Zhao W. Zhuang S. Qi X.-R. (2011). Comparative study of the *in vitro* and *in vivo* characteristics of cationic and neutral liposomes. Int. J. Nanomed. 6, 3087–3098. 10.2147/IJN.S25399 22163162 PMC3235029

[B226] Zhou M. Huang H. Wang D. Lu H. Chen J. Chai Z.. (2019). Light-triggered PEGylation/dePEGylation of the nanocarriers for enhanced tumor penetration. Nano Lett. 19, 3671–3675. 10.1021/acs.nanolett.9b00737 31062980

[B227] Zhu L. Torchilin V. P. (2013). Stimulus-responsive nanopreparations for tumor targeting. Integr. Biol. 5, 96–107. 10.1039/c2ib20135f 22869005 PMC3521849

[B228] Zinger A. Koren L. Adir O. Poley M. Alyan M. Yaari Z.. (2019). Collagenase nanoparticles enhance the penetration of drugs into pancreatic tumors. ACS Nano. 13, 11008–11021. 10.1021/acsnano.9b02395 31503443 PMC6837877

